# EphB1‐Mediated Transient Blood‐Brain Barrier Opening Facilitates a Ferritin‐Based Nanotherapeutic for Alzheimer's Disease

**DOI:** 10.1002/advs.76480

**Published:** 2026-07-06

**Authors:** Shilin Wen, Jingjing Gao, Zhixian Wang, Qiumin Ma, Xiao‐Ling Xu, Jianer Chen

**Affiliations:** ^1^ Department of Rehabilitation The Third Affiliated Hospital of Zhejiang Chinese Medical University Hangzhou Zhejiang China; ^2^ Shulan International Medical College Zhejiang Shuren University Hangzhou China; ^3^ The Third Clinical Medical College Zhejiang Chinese Medical University Hangzhou China; ^4^ Department of Neurorehabilitation Zhejiang Rehabilitation Medical Center Hangzhou China; ^5^ Department of Rehabilitation Affiliated Nanhua Hospital University of South China Hunan Province Hengyang China

**Keywords:** Alzheimer's disease, apoferritin, blood‐brain barrier, EphB1 receptor, nanotherapeutics, targeted drug delivery

## Abstract

The treatment of Alzheimer's disease (AD) is severely hampered by the blood‐brain barrier (BBB), which limits the delivery of therapeutic agents like donepezil (DPZ), an acetylcholinesterase inhibitor. While DPZ has multi‐faceted benefits, its clinical efficacy is constrained by poor BBB penetration, requiring high doses that lead to significant side effects. To overcome this, we developed a brain‐targeted nanotherapeutic utilizing apoferritin (AFn) nanoparticles loaded with DPZ (AFn‐DPZ). We demonstrate that this platform, by binding to the EphB1 receptor on the blood–brain barrier, enables transient and reversible opening of the blood–brain barrier, thereby facilitating efficient and targeted drug delivery. Following intravenous administration in an AD mouse model, AFn‐DPZ exhibited enhanced brain accumulation and sustained release of DPZ. This targeted delivery inhibited acetylcholinesterase activity, reduced amyloid plaque burden, alleviated neuroinflammation, attenuated oxidative damage, restored mitochondrial function, and upregulated the expression of brain‐derived neurotrophic factor (BDNF). Consequently, AFn‐DPZ treatment significantly improved cognitive performance compared to free DPZ. Our findings establish EphB1‐mediated facilitation of BBB traversal as a promising strategy for enhancing nanotherapeutic delivery to the brain, offering a potent approach to address the complex pathology of AD.

## Graphical abstract

We constructed a brain‐targeted AFn‐DPZ nanodrug delivery system and identified EphB1 as its receptor on the blood‐brain barrier (BBB). This system facilitates drug transport across the BBB and thereby ameliorates cognitive impairment in Alzheimer's disease.

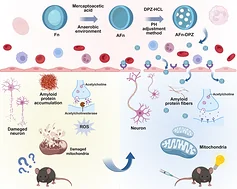

## 1 Introduction

AD is the most prevalent cause of dementia, representing a major global public health challenge. It is estimated that approximately 50 million individuals worldwide are currently affected, and this number is projected to exceed 150 million by 2050 as populations age [1]. AD is characterized by progressive cognitive decline, memory impairment, and functional deterioration, ultimately leading to complete dependence and death [2]. The disease imposes not only an enormous social and emotional toll but also a substantial economic burden, with global annual costs exceeding one trillion U.S. dollars [3]. Despite decades of investigation, there is still no curative therapy, and existing interventions primarily aim to alleviate symptoms rather than alter disease progression [4].

The pathological hallmarks of AD include extracellular β‐amyloid (Aβ) plaques, intracellular neurofibrillary tangles composed of hyperphosphorylated tau, neuroinflammation, oxidative stress, and marked deficits in cholinergic neurotransmission [5]. The loss of acetylcholine (ACh), a key neurotransmitter in learning and memory, is a central contributor to cognitive dysfunction [6]. Consequently, cholinesterase inhibitors remain the mainstay of pharmacological therapy [7]. Among them, DPZ is the most widely used due to its selectivity for acetylcholinesterase (AChE) and its ability to enhance synaptic ACh levels, thereby improving cognitive performance in mild to severe AD [8]. In addition to its cholinergic activity, DPZ exhibits pleiotropic neuroprotective effects, including suppression of Aβ aggregation, mitigation of neuroinflammatory cytokine release, and reduction of oxidative stress [9, 10, 11, 12]. However, the clinical efficacy of DPZ remains limited, primarily due to its poor penetration of the BBB. Owing to extensive first‐pass metabolism and low bioavailability, high systemic doses are required to achieve minimal therapeutic concentrations in the brain, leading to adverse effects such as nausea, gastrointestinal discomfort, and muscle cramps [13, 14, 15]. Thus, overcoming the BBB remains a key obstacle in maximizing the therapeutic potential of DPZ and other neuroprotective agents.

The BBB, a tightly regulated interface formed by endothelial cells, astrocytes, and pericytes, maintains central nervous system (CNS) homeostasis by restricting the entry of most macromolecules and xenobiotics [16]. While essential for neural protection, this barrier severely limits the delivery of therapeutic compounds to the brain [17]. To address this challenge, nanoparticle‐based drug delivery systems have emerged as a promising strategy. Nanocarriers can be engineered to encapsulate drugs, prolong their circulation, and facilitate targeted transport across the BBB [18]. A variety of nanomaterials, including liposomes [19], polymeric nanoparticles [20], inorganic particles [21], and metal–organic frameworks [22], have been investigated for brain delivery. However, many synthetic nanoparticles suffer from low biocompatibility, poor biodegradability, and potential toxicity, impeding their clinical translation [23].

In contrast, biologically derived nanocarriers such as ferritin offer distinct advantages. Ferritin, a ubiquitous iron storage protein composed of 24 subunits, self‐assembles into a spherical nanocage with an outer diameter of ∼12 nm and an internal cavity suitable for encapsulating therapeutic molecules [24]. Its natural origin, high stability, excellent biocompatibility, and capacity for surface modification make it an attractive candidate for biomedical applications [25, 26, 27]. Importantly, ferritin has been reported to cross the BBB through receptor‐mediated transcytosis, raising great interest in its use for CNS drug delivery [28, 29]. Specifically, the naturally occurring AFn nanocage has attracted considerable attention. Yet, the molecular mechanism behind its transport remains highly debated. While early studies suggested transferrin receptor 1 (TfR1) mediates this process, recent work by Yan et al. demonstrated that AFn lacks binding affinity for mouse TfR1. Paradoxically, numerous studies confirm that AFn efficiently traverses the BBB in vivo, indicating that an alternative receptor must be responsible [30, 31].

To resolve this conflict and advance the clinical application of AFn, we developed an apoferritin‐based nanotherapeutic encapsulating donepezil (AFn–DPZ) and systematically investigated its interaction with brain endothelial receptors. Using Octet biolayer interferometry, we identified EphB1, a member of the Eph receptor tyrosine kinase family, as a specific, high‐affinity binding partner for AFn. EphB1 is known to regulate vascular permeability and actin dynamics, a process integral to the structural integrity of endothelial tight junctions. We found that AFn binding to EphB1 triggers a reversible actin rearrangement, resulting in transient BBB modulation that facilitates efficient drug transport without compromising long‐term barrier integrity. In an AD mouse model, intravenous administration of AFn–DPZ led to markedly enhanced brain accumulation of DPZ compared to the free drug. This targeted delivery inhibited acetylcholinesterase activity, reduced amyloid plaque burden, alleviated neuroinflammation, attenuated oxidative damage, restored mitochondrial function, and upregulated the expression of brain‐derived neurotrophic factor (BDNF). Consequently, AFn–DPZ treatment significantly improved cognitive performance, demonstrating superior therapeutic efficacy over conventional formulations. Our findings not only establish the mechanism by which EphB1 mediates the transport of AFn across the BBB, but also reveal its potential as a controllable pathway for enhancing the delivery of nanotherapeutics to the brain Figure 1.

**FIGURE 1:**
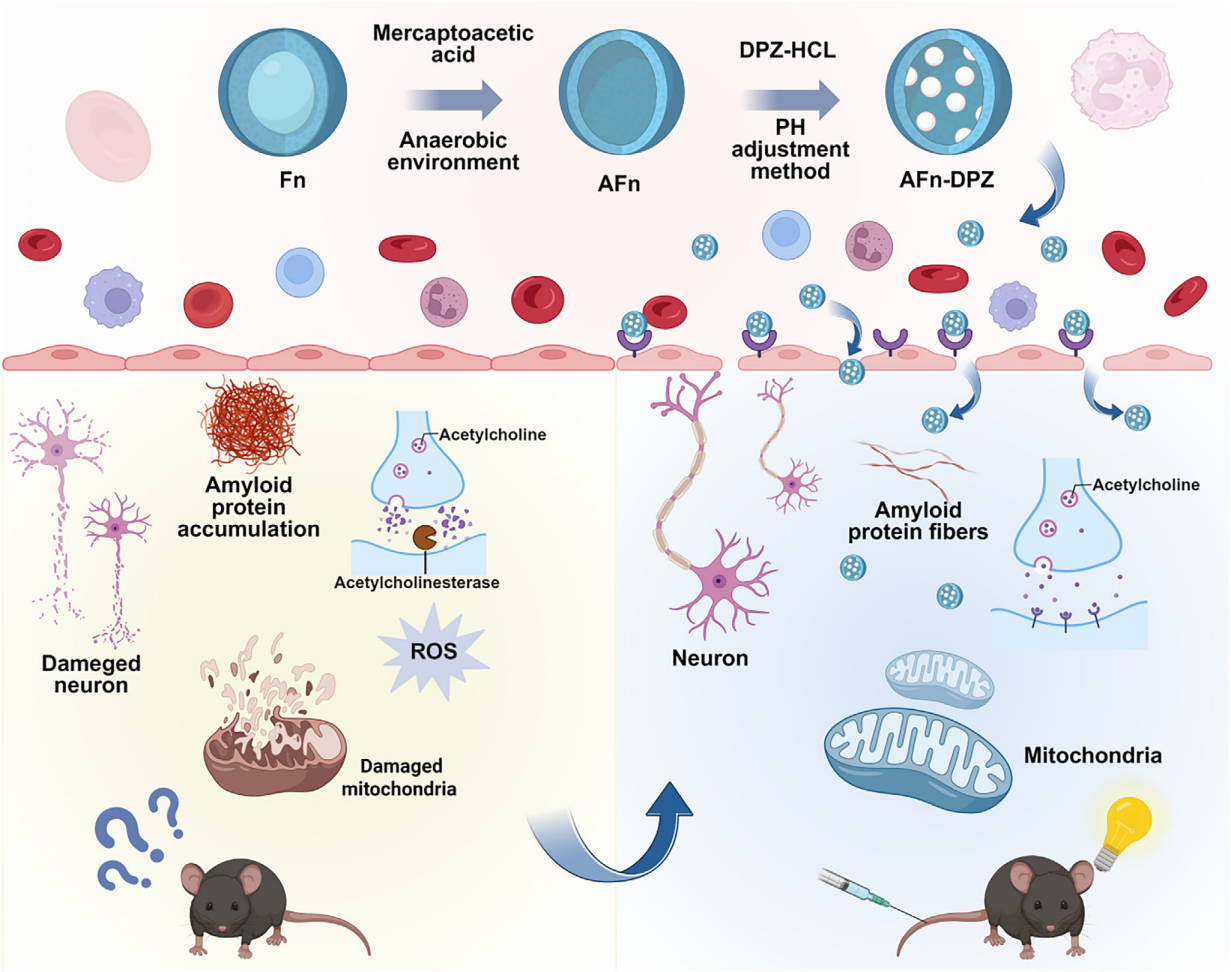
Schematic overview of the preparation of AFn‐DPZ and its application for improving AD pathology. Created with Biorender.

## 2 Materials and Methods

### 2.1 Materials, Cell Lines, and Animals

Donepezil HCl was purchased from Aladdin (Shanghai, China). Ferritin from equine spleen was obtained from Sigma–Aldrich (St. Louis, MO, USA). Dialysis bags (MWCO: 7000 Da) were sourced from Shanghai Yuanye Bio‐Technology Co., Ltd. (Shanghai, China). Amicon Ultra centrifugal filters (30 kDa) were procured from Merck Millipore Ltd. (Cork, Ireland). FITC‐dextran and FITC were supplied by Shanghai Yuanye Bio‐Technology Co., Ltd. (Shanghai, China). Aβ25‐35, DIR and FITC were purchased from MedChemExpress (Monmouth Junction, NJ, USA). EphB1‐IN‐1 were purchased from MedChemExpress (Monmouth Junction, NJ, USA). The AChE ELISA Kit was acquired from Wuhan Feiyue Biotechnology Co., Ltd. (Wuhan, China). The BDNF ELISA Kit was acquired from Wuhan Feiyue Biotechnology Co., Ltd. (Wuhan, China).The Aβ1‐42 ELISA Kit was obtained from Sangon Biotech (Shanghai, China). IL‐1β and TNF‐α ELISA Kits were provided by Boster Biological Technology, Ltd. (Wuhan, China). Transwell inserts were sourced from Corning Inc. (Corning, NY, USA). JC‐1, Reactive Oxygen Species Assay Kit, Actin‐Tracker Green‐488, and the BCA Protein Assay Kit were all purchased from Beyotime Biotechnology (Nanjing, China). A Cell Counting Kit‐8 (CCK‐8) was procured from MeilunBio (Dalian, China). Beta Amyloid Polyclonal Antibody was obtained from Thermo Fisher Scientific, and Anti‐Rabbit IgG (H+L) Antibody was purchased from SeraCare Life Sciences.

The bEnd.3 and SH‐SY5Y cell lines were acquired from Procell Life Science & Technology Co., Ltd. (Wuhan, China). bEnd.3 cells were maintained in specialized bEnd.3 cell culture medium, while SH‐SY5Y cells were cultured in their corresponding specific medium, both supplied by Procell Life Science & Technology Co., Ltd. (Wuhan, China). All cell lines were incubated at 37°C in a humidified atmosphere containing 5% CO_2_.

Six‐month‐old male APP/PS1 mice and wild‐type (WT) mice were supplied by Taizhou Huachuang Biotechnology Co., Ltd. The APP/PS1 transgenic Alzheimer's disease mouse model carries human amyloid precursor protein (APP) and the Swedish mutation of human presenilin 1 (PS1) genes. All animals were housed under controlled conditions with a temperature of 22 ± 2°C, humidity of 45% ± 10%, and a 12‐h light/dark cycle. The animal study protocol was approved by the Experimental Animal Management and Ethics Committee of Zhejiang Chinese Medical University (Approval No.: IACUC‐202504‐07).

### 2.2 Preparation and Characterization of AFn Nanocages and AFn‐DPZ

A 0.1 mol/L sodium acetate‐acetic acid buffer solution was prepared. 70% thioglycolic acid was added to the buffer at a volume percentage of 0.145%, and a nitrogen atmosphere was maintained throughout the process to ensure an oxygen‐free environment. A 10 mL dispersion of ferritin rich in H and L chains was placed into a dialysis bag and immersed in the sodium acetate‐acetic acid buffer under continuous nitrogen bubbling with stirring for dialysis.After dialysis, the liquid inside the dialysis bag was collected and sterilized by filtration through a 0.22 µm aqueous filter. The protein concentration was determined using a BCA protein quantification kit, and the sample was stored in a refrigerator for subsequent use.The size distribution and zeta potential of the prepared AFn were analyzed using a particle size analyzer. The morphology of AFn was examined by TEM. The iron ion content in ferritin, before and after iron removal, was detected using inductively coupled plasma‐mass spectrometry (ICP‐MS).

To prepare the AFn‐DPZ complex, 20 µL of 10 mg/mL donepezil hydrochloride (DPZ‐HCl) solution was added to 1 mL of 1 mg/mL AFn solution, resulting in a mass ratio of 5:1 (AFn:DPZ). The pH‐mediated drug loading method was employed. The mixture was acidified to pH 2.0 using 1 m HCl and stirred overnight at 4°C to facilitate protein disassembly into subunits. Subsequently, the pH was readjusted to 7.0–7.4 with 1 m NaOH and stirring continued at 4°C for 2 h to promote protein reassembly and drug encapsulation.Free, unencapsulated DPZ was removed by three cycles of ultrafiltration using a 30 kDa molecular weight cut‐off (MWCO) filter at 4000 rpm for 10 min per cycle. The final AFn‐DPZ nanocarriers were characterized for their size distribution and zeta potential using a particle size analyzer. Morphological examination was performed by transmission electron microscopy (TEM).

The stability of AFn and AFn‐DPZ was evaluated by storing the samples at 4°C for 15 days. Changes in particle size and zeta potential were monitored using a particle size analyzer at predetermined time points to assess their colloidal stability over time.

#### 2.2.1 Hemolysis Assay

Whole blood (1 mL) was collected from healthy mice and transferred into an anticoagulant tube. The tube was gently inverted to prevent coagulation. The blood was then diluted with 5 mL of 0.9% sodium chloride solution and mixed thoroughly. The mixture was centrifuged at 1000 rpm for 15 min. After centrifugation, the supernatant was carefully removed using a pipette. The pelleted red blood cells (RBCs) were resuspended in 5 mL of 0.9% sodium chloride solution and centrifuged again under the same conditions. This washing procedure was repeated three times or until the supernatant became clear and colorless. Finally, the purified RBCs were resuspended in 0.9% sodium chloride solution to prepare a 2% (v/v) suspension.For the hemolysis assay, the RBC suspension was incubated with the following test samples: negative control (saline), positive control (deionized water), free DPZ, AFn, and AFn‐DPZ. The mixtures were then centrifuged at 3000 rpm for 15 min. The hemolytic effect in each tube was documented photographically. The absorbance of the supernatant was measured at 541 nm, and the hemolysis rate was calculated.

#### 2.2.2 In Vitro Release Study

The in vitro release profiles of free DPZ and AFn‐DPZ were investigated using a dialysis method. A 1 mL volume of each sample was loaded into a dialysis bag (MWCO: 7000 Da). The bag was then immersed in PBS release medium and dialyzed under sink conditions in a constant temperature shaker at 37°C and 60 rpm.At predetermined time intervals, aliquots of the external PBS medium were collected and analyzed using a microplate reader. The percentage of drug released at each time point was calculated using an established formula.

### 2.3 Blood‐Brain Barrier Transport and Neuron Targeting

#### 2.3.1 Molecular Fishing Assay

bEnd.3 cells were lysed using an appropriate volume of RIPA lysis buffer supplemented with PMSF. The mixture was gently agitated and incubated on ice for 30 min. Subsequently, the lysate was centrifuged at 12,000 rpm for 20 min to collect the supernatant. The resulting protein extract was subjected to pull‐down assays with AFn using an OCTET molecular interaction system. The captured protein complexes were then analyzed by mass spectrometry.

#### 2.3.2 Molecular Docking Analysis

The AlphaFold structures corresponding to UniProt accession numbers Q8CBF3 and P02791 were retrieved from the UniProt database. Molecular docking was performed using the HDOCK online server. The model with the highest docking score was selected and visualized using PyMOL for further analysis.

#### 2.3.3 Phalloidin Staining

AFn was added to bEnd.3 cells with established tight junctions and incubated for 1 h and 3 h. Briefly, the cells were washed three times with PBS (5 min per wash), fixed with 4% paraformaldehyde for 10 min, and then washed again three times with PBS (5 min per wash). Subsequently, the cells were permeabilized with an immuno‐permeabilization solution for 10 min, followed by another three PBS washes (5 min per wash). Then, 100 µL of the phalloidin working solution (diluted 1:100 in PBS containing 10% FBS) was added to each dish, and the cells were incubated at room temperature protected from light for 50 min. After incubation, the cells were washed three times with PBS (5 min per wash). Nuclei were counterstained with DAPI for 5–10 min, followed by three quick PBS washes. Images were acquired using a confocal microscope.

#### 2.3.4 Immunofluorescence Staining and Confocal Imaging

AFn was added to bEnd.3 cells with established tight junctions and incubated for 1 h and 3 h. The cells were then gently rinsed three times with ice‐cold PBS and fixed with ice‐cold methanol at −20°C for 15 min. After fixation, the cells were washed again with PBS and blocked with PBS containing 10% FBS at room temperature for 1 h. The blocking solution was removed, and Occludin Monoclonal antibody (1:200 dilution, 66378‐1‐Ig, Proteintech) was added directly, followed by incubation at 4°C overnight. The next day, the primary antibody was discarded, and the cells were thoroughly washed three times with PBS. Subsequently, the cells were incubated in the dark at room temperature with Alexa Fluor 488 Goat Anti‐mouse IgG (H+L) (1:200 dilution, BK‐M488‐100ul, Bioker) for 1 h, followed by three thorough washes with PBS. Finally, the cells were incubated with DAPI for 10 min, washed three times thoroughly with PBS, and observed and imaged under a confocal laser scanning microscope.

#### 2.3.5 In Vivo Biodistribution Study

The fluorescent dye DIR was employed to evaluate the in vivo distribution of the formulation. Mice were randomly divided into two groups and administered AFn‐DIR or DIR alone via tail vein injection. Brain tissues were harvested at predetermined time points (1, 3, 9, and 24 h) and imaged using an in vivo imaging system. Brain tissues collected at the 3‐h time point were subsequently fixed, embedded, sectioned, and examined under a confocal microscope to visualize fluorescence distribution.

#### 2.3.6 Transport Study in an In Vitro BBB Cell Model

SH‐SY5Y cells were seeded in confocal dishes at a density of 1×10^5^ cells per dish and treated with FITC‐labeled AFn. The cellular uptake of AFn‐FITC was monitored in vitro at various time points using confocal microscopy.

To assess potential penetration and targeting effects, a bEnd.3/SH‐SY5Y co‐culture model was established. bEnd.3 cells were seeded in the upper chamber of a Transwell insert and cultured for several days to form a confluent monolayer. The integrity of the monolayer was monitored daily by measuring the transendothelial electrical resistance (TEER) using an EVOM^2^ volt‐ohm meter. Only inserts with a TEER value exceeding 100 Ω·cm^2^ were used for the in vitro BBB permeability assay. The integrity of the BBB model was further validated by performing a leakage assay with 4 kDa FITC‐dextran. Subsequently, a BBB transcytosis assay was conducted by adding FITC‐labeled AFn to the specific medium in the upper chamber. The internalization of AFn‐FITC in the SH‐SY5Y cells located in the basolateral compartment was visualized by confocal microscopy at different time points.

#### 2.3.7 Liquid Chromatography‐Tandem Mass Spectrometry (LC‐MS/MS)

To assess the ability of AFn to deliver DPZ across the BBB, APP/PS1 mice were injected with free DPZ and AFn‐DPZ. At various time points (0.25, 2, 3 and 24 h), mice were perfused, and brain tissues were collected and snap‐frozen in liquid nitrogen. Approximately 0.2 g of brain tissue was homogenized, and 200 µL of PBS solution (0.01 m, pH 7.40) was added. The mixture was vortexed to obtain a homogenate. Then, 100 µL of the homogenate was mixed with 10 µL of 0.1 m NaOH solution for alkalization, followed by the addition of 1 mL of ethyl acetate for extraction. After vortexing and centrifugation at 10,000 rpm for 5 min, the supernatant was collected and concentrated to dryness by centrifugation. The residue was reconstituted in 80 µL of 50% methanol in water and then subjected to LC‐MS/MS analysis.

### 2.4 In Vitro Anti‐AD Efficacy Study

#### 2.4.1 Biocompatibility Assessment of AFn and AFn‐DPZ by CCK‐8 Assay

SH‐SY5Y cells were seeded in 96‐well plates at a density of 1×10^4^ cells per well and cultured overnight. The cells were then treated with various concentrations of AFn or AFn‐DPZ for 24 h. Cytotoxicity of AFn and AFn‐DPZ toward SH‐SY5Y cells was assessed using the CCK‐8 assay, and the absorbance at 450 nm was measured with a microplate reader.

#### 2.4.2 Dose Optimization of Aβ_25_‑_35_ for AD Cell Model Induction and Subsequent DPZ Efficacy Screening

SH‐SY5Y cells were seeded in 96‐well plates at a density of 1×10^4^ cells per well and cultured overnight. The cells were then treated with various concentrations of Aβ**_25_‑_35_** for 12 h. The cytotoxicity induced by Aβ25‐35 was assessed using the CCK‐8 assay, and the absorbance at 450 nm was measured with a microplate reader.

In a separate experiment, SH‐SY5Y cells were similarly seeded and cultured overnight. They were then co‐treated with DPZ and Aβ**_25_‑_35_** for 12 h. The protective effect of DPZ against Aβ_25_‑_35_‐induced cytotoxicity in SH‐SY5Y cells was evaluated using the CCK‐8 assay, followed by measurement of the absorbance at 450 nm with a microplate reader.

#### 2.4.3 Effects of AFn‐DPZ on ROS and Mitochondrial Membrane Potential (MMP) in Aβ_25_‑_35_‐Induced AD Cellular Models

SH‐SY5Y cells were seeded in confocal dishes at a density of 1×10^5^ cells per dish and cultured overnight. The cells were then treated with 60 µM Aβ_25_‑_35_ to establish the model, while simultaneously co‐treated with DPZ, AFn, or AFn‐DPZ. After 12 h of incubation, the cells were subjected to JC‐1 staining and DCFH‐DA staining, followed by imaging with a confocal microscope.

### 2.5 In Vivo Anti‐AD Efficacy Evaluation

Male APP/PS1 mice were randomly allocated into four groups: APP/PS1 group, APP/PS1+DPZ group, APP/PS1+AFn group, and APP/PS1+AFn‐DPZ group. wild‐type (WT) mice were used as the normal control group. The APP/PS1 group received intravenous tail vein injections of normal saline every other day. The APP/PS1+DPZ, APP/PS1+AFn, and APP/PS1+AFn‐DPZ groups were administered their respective treatments via tail vein injection on the same every‐other‐day schedule. All treatments continued for a period of two weeks.

In the present study, the sample size for each group in the behavioral experiments was four mice (n = 4). Inclusion criteria were qPCR‐confirmed genotype (APP/PS1 or WT mice), age of six months, male, and the absence of apparent morphological or locomotor abnormalities. Exclusion criteria comprised the development of severe health issues during the testing period, an inability to swim, or missing/aberrant data. Mice were randomly assigned to experimental groups using a random number table. A double‐blind design was implemented throughout the experiments, meaning that both the investigator performing the procedures and the analyst responsible for data evaluation were unaware of the group allocation.

#### 2.5.1 Nesting Behavior Test

Upon completion of the treatment regimen, a nest‐building test was conducted to assess the social behavior of the mice. In this test, each mouse was individually housed in a separate cage, with two identical Nestlet pads (5 cm × 5 cm) placed in the same corner of the cage. The condition of the nesting materials and nest construction were photographed and recorded at 24 and 72 h. The nesting performance was subsequently scored based on established criteria from previous studies.

#### 2.5.2 Morris Water Maze Test

The mice were placed in a cylindrical pool with a diameter of 120 cm, filled with water to a depth of 50 cm, and maintained at a temperature of 25 ± 2°C. The pool was divided into four quadrants, each marked with a distinct geometric shape. A circular platform, 10 cm in diameter and 30 cm in height, was positioned in the center of the fourth quadrant, submerged 1 cm below the water surface.The experiment consisted of three phases: one day of adaptive training, five days of place navigation, and one day of spatial probe testing. During the place navigation trials, each mouse was released into the water facing the pool wall from one of four different starting points. The escape latency, defined as the time taken to locate the hidden platform, was recorded for each trial. If a mouse found the platform within 60 s, the system automatically recorded the latency. If it failed to locate the platform within the allotted time, the mouse was gently guided to the platform and allowed to remain there for 20 s. Following the place navigation phase, the platform was removed for the spatial probe test. During this phase, the number of platform crossings and the time spent in the target quadrant were automatically recorded by the system over a 60‐s period.

#### 2.5.3 Special Staining and Immunohistochemistry

On the day following the completion of behavioral tests, all mice were subjected to blood collection via eyeball extraction and then sacrificed for brain tissue harvesting and subsequent histological staining. The brain tissues were dissected, fixed in 4% paraformaldehyde, and sectioned into 5 µm slices. These sections were stained with Congo red and Nissl dye according to their respective standard protocols.

For immunohistochemical analysis, the brain sections were incubated with a blocking solution (PBS containing 5% BSA and 5% bovine serum) for 2 h. After washing, the sections were incubated with primary antibodies at 4°C overnight. Following PBS washes, the sections were treated with corresponding secondary antibodies and incubated at room temperature for 1 h. Images were captured using a microscope and subsequently subjected to semi‐quantitative analysis using ImageJ software.

#### 2.5.4 In Vivo Biocompatibility Evaluation

Blood samples were collected via eyeball extraction to obtain whole blood and serum for complete blood count and assessment of hepatic and renal function. The heart, liver, spleen, lungs, and kidneys were harvested, embedded in paraffin, and sectioned at a thickness of 4 µm. Following deparaffinization, the sections were subjected to hematoxylin and eosin (H&E) staining. Histopathological changes were then examined under a microscope.

#### 2.5.5 Sample Preparation and ELISA

The mouse hippocampi were frozen in liquid nitrogen and homogenized in RIPA lysis buffer supplemented with PMSF at a 100:1 ratio. The homogenates were centrifuged at 12,000 rpm and 4°C to collect the supernatant. According to the manufacturer's protocols, the concentrations of AChE, Aβ1‐42, IL‐1β, and TNF‐α were quantified using respective ELISA kits. Absorbance was measured at a wavelength of 450 nm with a microplate reader.

#### 2.5.6 Brain Proteomics

Hippocampal samples were first retrieved from ‐80°C storage and pulverized into powder in liquid nitrogen. An appropriate amount of the powdered tissue was then transferred to a 1.5 mL centrifuge tube and lysed using a buffer containing 8 M urea, 1 mM PMSF, and 2 mM EDTA, followed by sonication for 5 min on ice. The lysate was centrifuged at 15,000 × g for 10 min at 4°C, and the supernatant was collected. Protein concentration was determined using a BCA assay kit (Beyotime Biotechnology, Shanghai, China). For each sample, 100 µg of protein was taken, and the volume was adjusted to 200 µL with 8 M urea. Reduction was performed by adding DTT to a final concentration of 5 mM and incubating at 37°C for 45 min, followed by alkylation in the dark at room temperature using iodoacetamide at a final concentration of 11 mM for 15 min. Subsequently, 800 µL of 25 mM ammonium bicarbonate and 2 µL of trypsin (Promega, V5280) were added, and the mixture was digested overnight at 37°C. The resulting peptides were acidified to pH 2–3 using 20% TFA and desalted using C18 stage tips (Millipore, Billerica, MA). Peptide concentration was measured using the Pierce Quantitative Peptide Assay (Thermo Fisher). Peptide samples were separated using a Vanquish Neo UHPLC nanoflow system. For data‐independent acquisition (DIA) analysis, chromatographic separation was performed on a nanoflow Vanquish Neo system (Thermo Scientific), and the eluted peptides were analyzed using an Orbitrap Astral mass spectrometer (Thermo Scientific) operating in DIA mode.

A comprehensive quality control procedure was implemented at both the peptide and protein levels to ensure data reliability and robustness. Quantitative proteomic analysis was conducted to identify differentially abundant proteins. Visualization of these proteins was achieved by generating volcano plots and heatmaps. Functional enrichment analysis of the differentially expressed proteins was subsequently performed, including Gene Ontology (GO) term classification and Kyoto Encyclopedia of Genes and Genomes (KEGG) pathway mapping.

### 2.6 Statistical Analysis

All statistical analyses and graph preparations were performed using GraphPad Prism software (version 10.0). The normality of the data was assessed using the Shapiro–Wilk (SW) test. Data following a normal distribution were presented as mean ± SD. Comparisons between two groups were conducted using the unpaired Student's t‐test. For comparisons among multiple groups, a one‐way analysis of variance (ANOVA) was used, followed by Tukey's multiple comparisons test. The sample size (n) for each experiment is indicated in the corresponding figure legends. A *p*‐value < 0.05 was considered statistically significant.

## 3 Results

### 3.1 Characteristics of AFn and AFn‐DPZ

As previously mentioned, the solution color of ferritin changed from brownish‐red to pale yellow after iron removal (Figure 2a). TEM revealed that apoferritin (AFn) exhibited a typical hollow cage‐like structure (Figure 2b), with a particle size of approximately 9.66 ± 0.68 nm (Figure 2c) and a zeta potential of about −11.94 ± 0.59 mV (Figure 2 g). The iron content was significantly reduced after deferration (Figure 2d), confirming the successful preparation of hollow cage‐structured apoferritin.Upon loading with DPZ, AFn‐DPZ displayed a filled interior structure under TEM (Figure 2e), with a particle size of about 11.23 ± 1.78 nm (Figure 2f) and a zeta potential of approximately −7.44 ± 0.30 mV (Figure 2 g). The encapsulation efficiency of AFn‐DPZ was 33.30% ± 1.08%, and the drug loading capacity was 14.27% ± 0.40% (Figure S1a). The particle sizes of both AFn and AFn‐DPZ showed minimal change on days 1, 10, and 15 (Figure 2 h), while the absolute zeta potential values gradually increased over time (Figure 2i), indicating improved stability of the formulations during storage.To investigate whether DPZ was successfully incorporated into AFn‐DPZ and to evaluate the drug release profile, the in vitro release behavior was studied. Free DPZ reached a cumulative release of over 80% within 12 h, whereas AFn‐DPZ achieved over 80% cumulative release after 96 h (Figure 2j), demonstrating that AFn effectively delayed the release of DPZ, which may contribute to a prolonged in vivo effect. Hemolysis assays were conducted as described above. The hemolysis rates of DPZ + RBCs, AFn + RBCs, and AFn‐DPZ + RBCs were all below 5% (Figure 2k, l), indicating that the formulations possess good hemocompatibility and are safe for potential biomedical applications.

**FIGURE 2:**
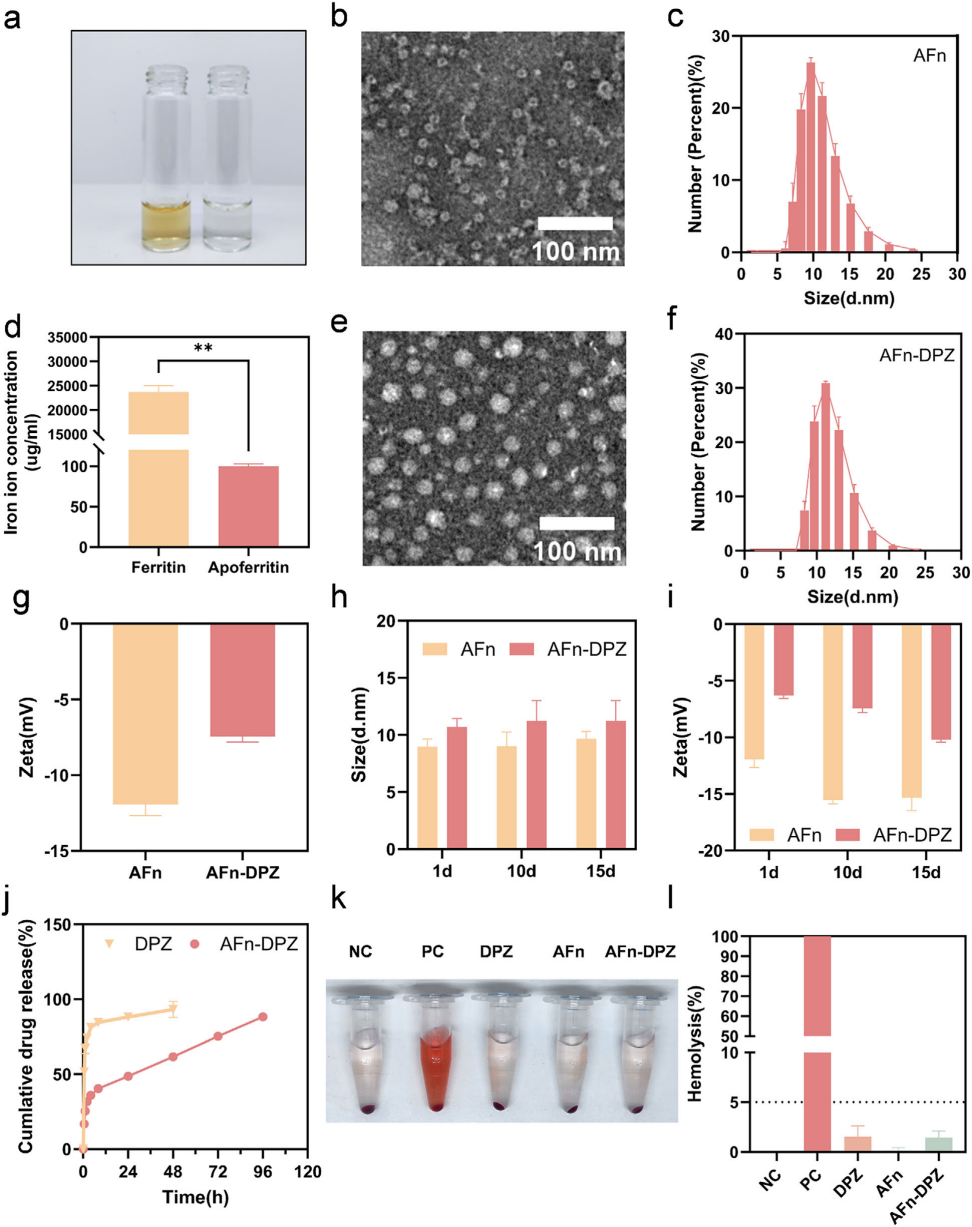
Characteristics of AFn Nanocages and AFn‐DPZ. (a) Schematic illustration of AFn before and after iron removal. (b) TEM images of AFn nanocages. (c) Particle size distribution of AFn (n = 3). (d) Iron content of ferritin before and after iron removal (n = 3). (e) TEM images of AFn‐DPZ. (f) Particle size distribution of AFn‐DPZ (n = 3). (g) Zeta potential of AFn and AFn‐DPZ (n = 3). (h) Particle size of AFn and AFn‐DPZ formulations on day 1, day 10, and day 15 (n = 3). (i) Zeta potential of AFn and AFn‐DPZ formulations on day 1, day 10, and day 15 (n = 3). (j) In vitro release profile of DPZ from AFn at pH 7.4 and 37°C (n = 3). (k) Representative images of hemolysis assays for each group. (l) Quantitative analysis of hemolysis rate for each group (n = 3).

### 3.2 Transport Across the Blood‐Brain Barrier and Intracellular Trafficking in SH‐SY5Y Cells

To identify specific receptors of AFn on the BBB, a molecular fishing assay was conducted (Figure 3a). Mass spectrometry analysis revealed that AFn specifically binds to EphB1 on the membrane of bEnd.3 cells (Figure 3b). Subsequently, molecular docking between AFn and EphB1 was performed using HDOCK (Figure 3c). Model 1 exhibited a Docking Score of −276.43, the lowest among all models, indicating the most favorable interaction energy. A Confidence Score of 0.9261 further confirmed the reliability of this docking model. In comparison, Models 2 and 3 showed slightly higher scores (−263.30 and −260.12, respectively) and lower Confidence Scores, suggesting inferior stability and credibility.

**FIGURE 3:**
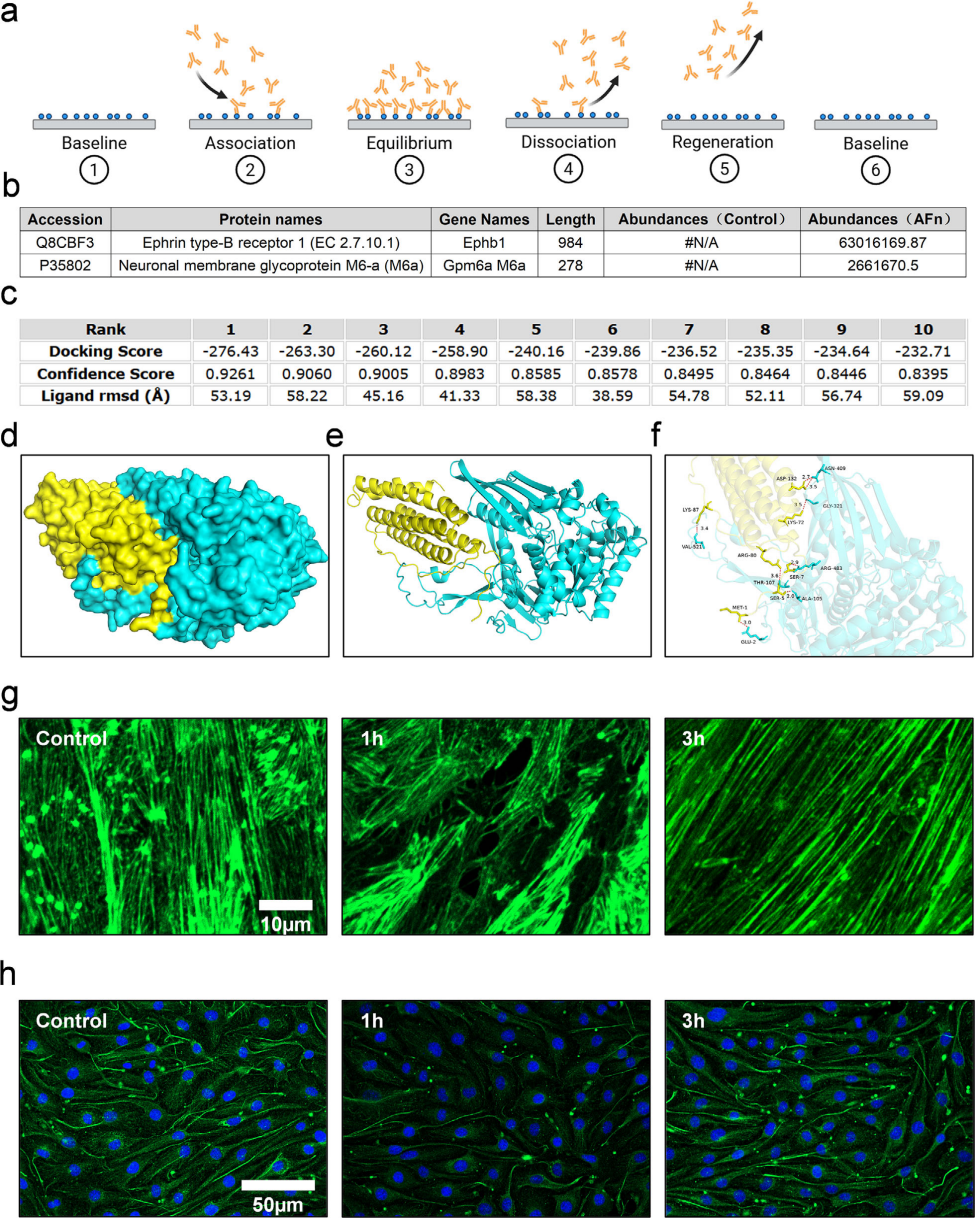
Identification and Validation of AFn Receptors on the BBB.(a) Schematic workflow of receptor fishing using AFn.(b) Mass spectrometric analysis following molecular fishing.(c) Molecular docking model of AFn with EphB1.(d,e,f) Visualization of the molecular docking between AFn and EphB1.(g) Phalloidin staining of bEnd.3 cells after 1 and 3 h incubation with AFn.(h) Representative immunofluorescence images showing the tight junction protein occludin in bEnd.3 cells following AFn incubation for 1 and 3 h.

For further validation, the top‐scoring model (Model 1, −276.43) was visualized using Pymol (Figure 3d–f).

For drugs to enter the brain, they must cross the BBB. EphB1 is a transmembrane protein expressed on bEnd.3 cells and serves as a key signaling receptor [32]. Eph receptors and their ligands are membrane‐bound molecules involved in intercellular communication, with well‐established roles in development. They induce cytoskeletal changes, promote actin filament crosslinking, and regulate actin cytoskeleton remodeling [33]. Moreover, EphB signaling facilitates cell retraction and repulsion via RhoA activation [34]. Studies have also shown that *EphB1^–/–^* mice exhibit significantly reduced endothelial permeability [35]. We added AFn to bEnd.3 cells that had already formed tight junctions and performed immunofluorescence experiments on the cytoskeletal protein actin (Figure 3g) and the tight junction protein Occludin (Figure 3h; Figure S2a). 1 h after adding AFn to bEnd.3 cells that had already formed tight junctions, we observed rearrangement of the cytoskeleton, accompanied by cell contraction. Concurrently, there was a reduction in the tight junction protein occludin and an increase in intercellular spacing. In contrast, after 3 h of AFn exposure, the bEnd.3 cells re‐established tight junction integrity. In the in vitro BBB model, TEER values were measured at 1 h and 3 h after AFn addition. A decrease in TEER was observed at 1 h, followed by an increase at 3 h (Figure S2b). These findings indicate that AFn binding to the EphB1 receptor transiently alters the cytoskeleton of bEnd.3 cells, promoting cell retraction, widening intercellular gaps, and enhancing permeability, thereby facilitating more efficient transport of AFn across the blood–brain barrier to act on neurons. In the in vitro BBB model, following treatment with an EphB1 inhibitor, FITC‐labeled AFn was added to the upper chamber. The experimental results demonstrated that the fluorescence intensity in the lower chamber was significantly stronger in the absence of the EphB1 inhibitor than in its presence (Figure S2c). Following EPHB1 inhibitor treatment, the trans‐BBB transport efficiency of AFn was significantly reduced. This result confirms that EphB1 serves as a key molecular basis for AFn to achieve efficient brain‐targeted delivery.

The ability of AFn to cross the BBB was further validated using both in vivo and in vitro models, as summarized in Figure 4.

**FIGURE 4:**
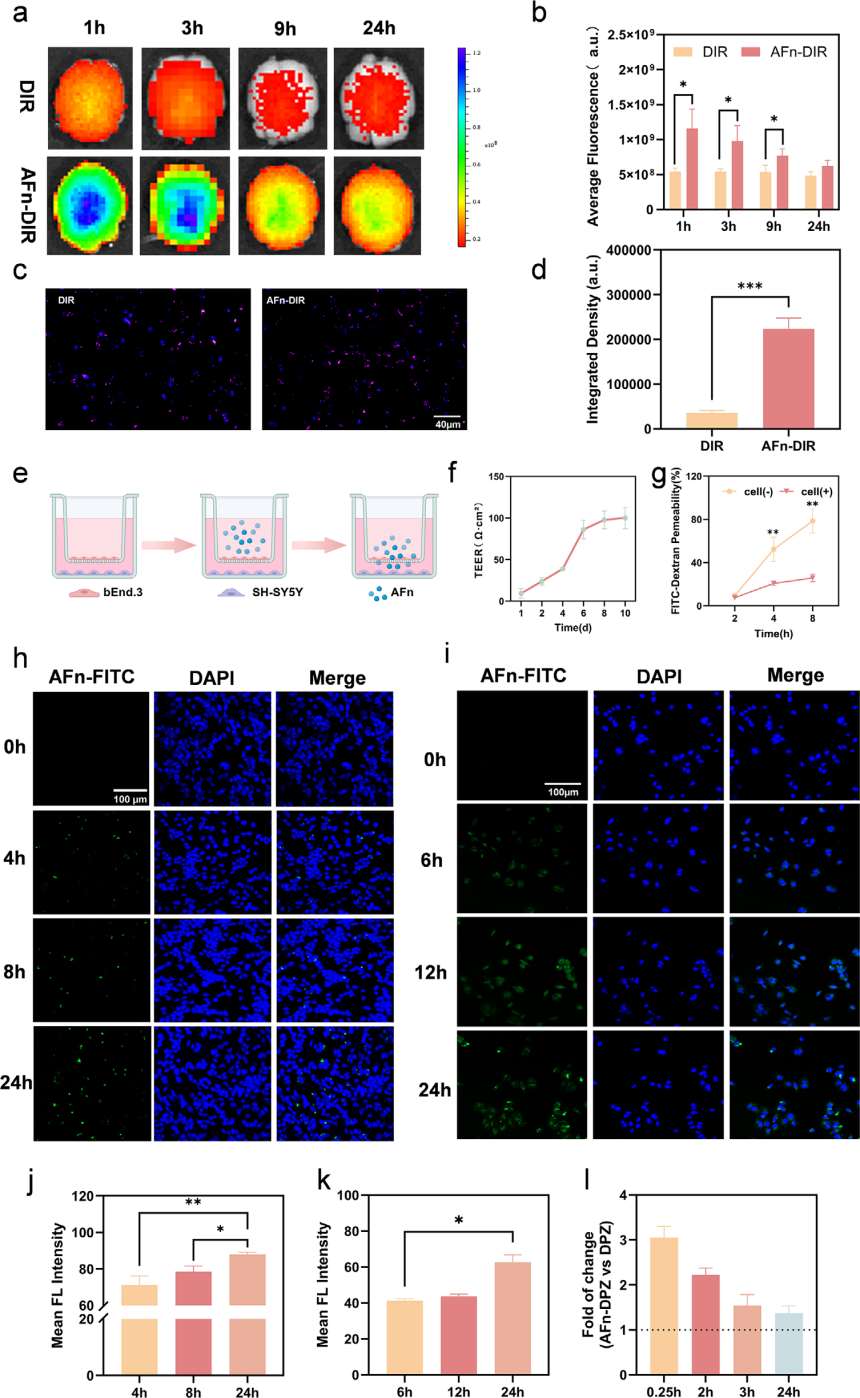
Investigation of AFn transport across the BBB in vitro and in vivo, and internalization of AFn‐FITC in SH‐SY5Y cells. (a) In vivo brain imaging of mice following intravenous injection of free DIR and AFn‐DIR. (b) Quantitative analysis of fluorescence intensity in the brain for free DIR and AFn‐DIR (n = 3). *p < 0.05 compared with the free DIR group. (c) CLSM images of brain sections collected at 3 h after tail vein injection of free DIR and AFn‐DIR. (d) Quantitative analysis of fluorescence intensity in brain sections for free DIR and AFn‐DIR (n = 3). ***p < 0.001 compared with the free DIR group. (e) Schematic diagram of AFn transport across a bEnd.3 cell monolayer in the Transwell model. (f) TEER values of the bEnd.3 monolayers prior to the experiment (n = 3). (g) Permeability of the cell‐free and bEnd.3‐seeded in vitro BBB models, assessed using 4 kDa FITC‐Dextran (n = 3). **p < 0.01 compared with the bEnd.3 cell‐seeded group. (h) CLSM images showing the internalization of AFn‐FITC in SH‐SY5Y cells following its transport across the bEnd.3 monolayer. (i) CLSM images showing the time‐dependent internalization of AFn‐FITC in SH‐SY5Y cells. (j) Quantitative analysis of AFn‐FITC internalization in SH‐SY5Y cells following its transport across the bEnd.3 monolayer (n = 3). **p < 0.01, ***p < 0.001 compared with the 24 h group. (k) Quantitative analysis of AFn‐FITC internalization in SH‐SY5Y cells at different time points (n = 3). *p < 0.05 compared with the 6 h group. (l) Using the free DPZ group as a reference, the relative drug multiples of the AFn‐DPZ group at different time points were calculated. The ratios at 0.25 h, 2 h, 3 h, and 24 h are presented (n = 3).

For in vivo evaluation, APP/PS1 mice were intravenously injected with AFn‐DIR or free DIR. 1 h post‐injection, strong fluorescence was observed in the brains of AFn‐DIR‐treated mice, indicating rapid brain delivery. Although the fluorescence intensity gradually declined over time, it remained significantly higher in the AFn‐DIR group than in the free DIR group at 1, 3, and 9 h (Figure 4a, b). This observation was further confirmed by CLSM of brain sections harvested at 3 h, which showed markedly stronger fluorescence in the AFn‐DIR group (Figure 4c, d). These results suggest that AFn can efficiently cross the BBB and target the brain, a property that may be beneficial for AD treatment.

Using an in vitro BBB model to evaluate AFn transport under simulated physiological conditions, a co‐culture system was established with bEnd.3 cells in the upper chamber and SH‐SY5Y cells in the lower chamber (Figure 4e). BBB formation was monitored by daily TEER measurements, which stabilized at approximately 100 Ω·cm^2^ after one week (Figure 4f). BBB integrity was further confirmed by a 4 kDa FITC‐dextran permeability assay (Figure 4g). After AFn‐FITC was added to the upper chamber, CLSM imaging revealed fluorescence in SH‐SY5Y cells in the lower chamber, which intensified over time (Figure 4h,j). Following the addition of DPZ and AFn‐DPZ to the upper chamber of the in vitro BBB model, the DPZ concentration in the lower chamber was measured by LC‐MS/MS, and the permeability coefficients across the in vitro BBB were calculated. The results showed that AFn‐DPZ exhibited a higher permeability than free DPZ (FigureS3a). Collectively, these experiments demonstrate that AFn can cross the BBB, enhance the drug permeability across the BBB, and promote the endocytosis of neuronal cells.

To assess the ability of AFn to deliver DPZ across the BBB, APP/PS1 mice were injected with free DPZ and AFn‐DPZ. The concentration of DPZ in mouse brain tissue was determined by LC‐MS/MS. The results showed that at 0.25 h, 2 h, and 3 h, the brain DPZ concentration in the AFn‐DPZ group was significantly higher than that in the free DPZ group (Table S1). Using the free DPZ group as a reference, the relative fold changes in brain DPZ concentration at 0.25, 2, 3, and 24 h for the AFn‐DPZ group were calculated to be approximately 3.05 ± 0.21, 2.22 ± 0.13, 1.54 ± 0.20, and 1.37 ± 0.13, respectively (Figure 4l). These results indicate that encapsulation of DPZ within the AFn nanocage enhances its brain accumulation efficiency, further demonstrating that the AFn delivery system can significantly improve the brain‐targeting ability and intracerebral bioavailability of DPZ. The plasma DPZ concentrations in APP/PS1 mice at different time points showed that in the free DPZ group, the plasma concentration was 45.10 ± 16.04 ng/mL at 0.25 h post‐administration, followed by a gradual decline. The plasma DPZ concentrations in the AFn‐DPZ group were lower than those in the free DPZ group at all time points; however, no significant differences were observed between the two groups (Table S2).

Finally, neuronal uptake was examined using in vitro cellular experiments. The results showed that SH‐SY5Y cells internalized AFn‐FITC in a time‐dependent manner, as evidenced by progressively increasing fluorescence intensity (Figure 4i,k). This finding suggests that AFn can not only cross the blood‐brain barrier but also be efficiently taken up by neuronal cells.

### 3.3 AFn‐DPZ Attenuates Neuronal Injury in Vitro

Mitochondrial impairment in neurons represents one of the earliest events in the pathological progression of AD, occurring even before clinical diagnosis. Previous studies have shown that Aβ_25_‑_35_ can induce mitochondrial dysfunction and reduce MMP, leading to diminished ROS clearance capacity [36, 37].To evaluate the antioxidant and neuroprotective effects of AFn‐DPZ, we first assessed the cytotoxicity of different formulations on SH‐SY5Y cells. Cell viability remained above 95% in both AFn and AFn‐DPZ groups across various concentrations (Figure 5a, b), indicating favorable biocompatibility.

**FIGURE 5:**
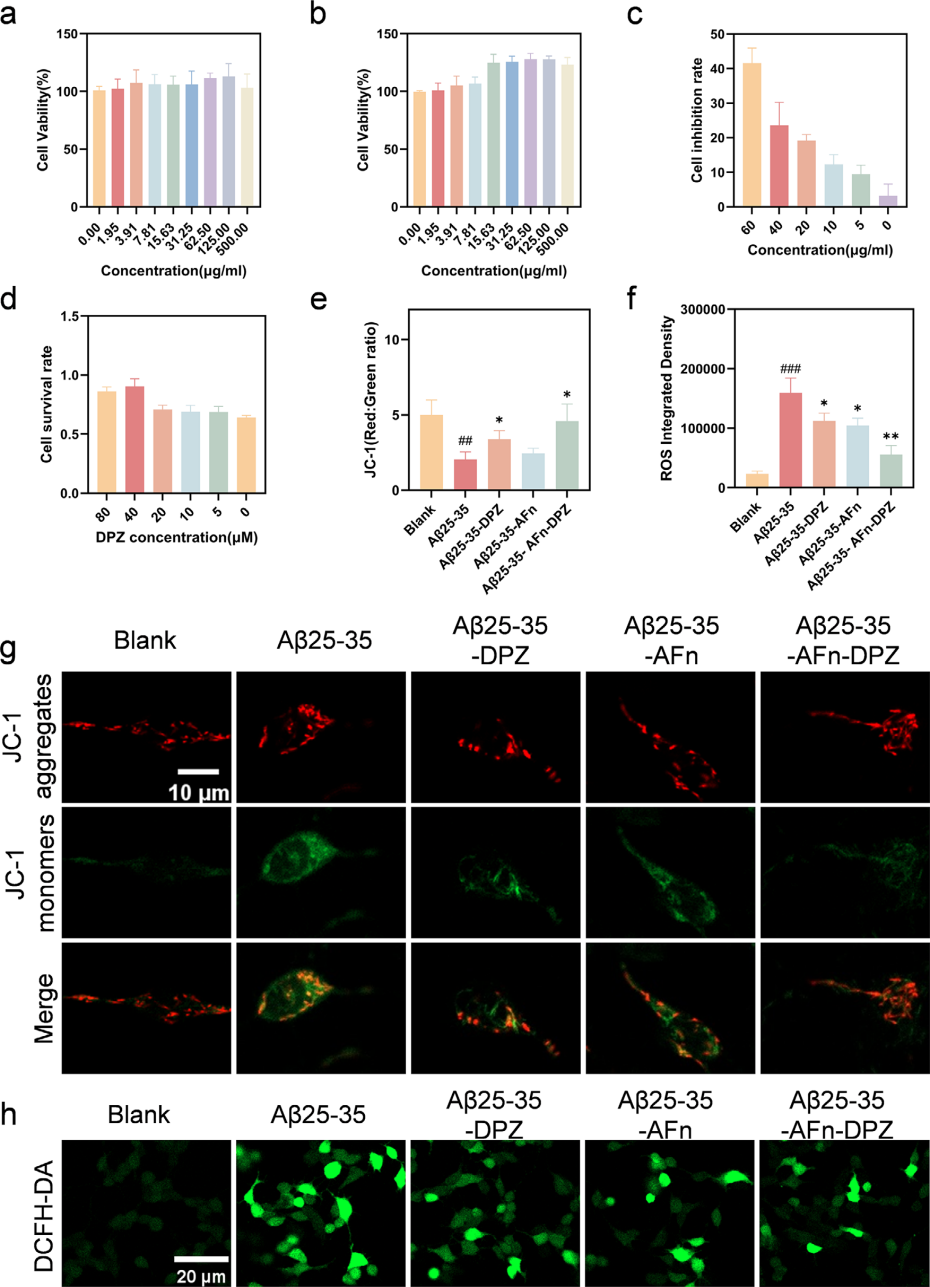
AFn‐DPZ protected neurons from Aβ_25_‑_35_ damage. (a) Cytotoxicity assessment in SH‐SY5Y cells treated with increasing concentrations of AFn (n = 3). (b) Cytotoxicity assessment in SH‐SY5Y cells treated with increasing concentrations of AFn‐DPZ (n = 3). (c) Viability of SH‐SY5Y cells treated with increasing concentrations of Aβ_25_‑_35_ (n = 3). (d) Viability of Aβ_25_‑_35_‐treated SH‐SY5Y cells following treatment with different concentrations of free DPZ (n = 3). (e) Quantitative analysis of MMP in different treatment groups after Aβ_25_‑_35_ insult, as measured by CLSM (n = 3). ## p < 0.01 compared with the blank group; * p < 0.05 compared with the Aβ_25_‑_35_ group. (f) Quantitative analysis of ROS generation in different treatment groups after Aβ_25_‑_35_ insult, as measured by CLSM (n = 3). ## p < 0.01 compared with the blank group; * p < 0.05 compared with the Aβ_25_‑_35_ group. (g) Representative CLSM images depicting changes in MMP across different treatment groups after Aβ_25_‑_35_ insult. (h) Representative CLSM images depicting ROS generation across different treatment groups after Aβ_25_‑_35_ insult. ### p < 0.001 compared with the blank group; * p < 0.05, ** p < 0.01 compared with the Aβ_25_‑_35_ group.

The Aβ_25_‑_35_ fragment, a core toxic peptide derived from full‐length Aβ, is known to induce oxidative stress, neurotoxicity, and inflammatory responses, and has been widely used to model Aβ‐related AD pathogenesis [38]. We thus established an AD‐like cellular model by treating SH‐SY5Y cells with Aβ_25_‑_35_. At concentrations of 60 µM or higher, Aβ_25_‑_35_ exhibited significant cytotoxicity (Figure 5c). A concentration of 60 µM was selected for subsequent modeling to ensure observable rescue effects. To determine an effective treatment concentration, we screened DPZ‐HCl in the Aβ_25_‑_35_‐induced AD model. As shown in Figure 5d, a DPZ‐HCl concentration of 40 µM resulted in approximately 97% cell viability. This concentration was therefore chosen for subsequent anti‐apoptotic and antioxidant experiments.

Aβ_25_‑_35_ is known to cause mitochondrial dysfunction and a consequent decrease in MMP [39]. We evaluated MMP in differently treated SH‐SY5Y cells using JC‐1 staining. Compared with untreated cells, those treated with Aβ_25_‑_35_ showed a lower red/green fluorescence ratio, indicating depolarized mitochondria and lower MMP. Treatment with AFn‐DPZ significantly attenuated this Aβ_25_‑_35_‐induced damage, as reflected by a higher red/green ratio compared to the Aβ_25_‑_35_‐only group (Figure 5e, g). Since decreased MMP has been linked to increased ROS production [40], we further assessed the antioxidant capacity of AFn‐DPZ in the Aβ_25_‑_35_‐induced AD model. Intracellular ROS levels were detected using the DCFH‐DA probe and confocal microscopy. Aβ_25_‑_35_ treatment markedly increased ROS generation, whereas all treatment groups showed reduced ROS levels. The lowest ROS level was observed in cells treated with 40 µM AFn‐DPZ (Figure 5f, h), suggesting a synergistic antioxidant effect between AFn and DPZ.

In summary, these results demonstrate that AFn‐DPZ effectively restores neuronal mitochondrial function, suppresses apoptosis, and alleviates oxidative stress damage caused by excessive ROS generation.

### 3.4 AFn‐DPZ Ameliorates Cognitive Decline in AD Model Mice

We employed transgenic APP/PS1 mice as an AD model and evaluated the neuroprotective efficacy of AFn‐DPZ in vivo. A series of behavioral tests were conducted to assess memory and cognitive function in AD mice following administration of various formulations.In the Morris water maze test, representative swimming paths for each group during the orientation navigation and spatial probe phases are shown in Figure 6a. Compared with WT mice, the APP/PS1 group exhibited impaired spatial learning, showing no significant reduction in the path length or time taken to first reach the target platform across training days. In contrast, mice in the treatment groups, particularly those treated with AFn‐DPZ, displayed a decreasing trend in swimming path length (Figure 6c). Mice with intact cognitive function demonstrated spatially oriented swimming behavior rather than random wandering, resulting in significantly shorter swimming distances and latencies than those in the APP/PS1 group (Figure 6d). During the spatial probe trial, APP/PS1 mice performed poorly. They exhibited longer path lengths to the target platform, spent considerably less time in the target quadrant, and showed fewer platform crossings compared to the WT group (Figure 6g). In contrast, AFn‐DPZ‐treated mice preferentially moved toward the original platform location, with shorter swimming paths to the target (Figure 6e), spent the longest time in the target quadrant (Figure 6f), and achieved the highest platform crossing frequency (Figure 6g). These results confirm that AFn‐DPZ exerted the most significant effect in alleviating spatial memory deficits in AD mice.

**FIGURE 6:**
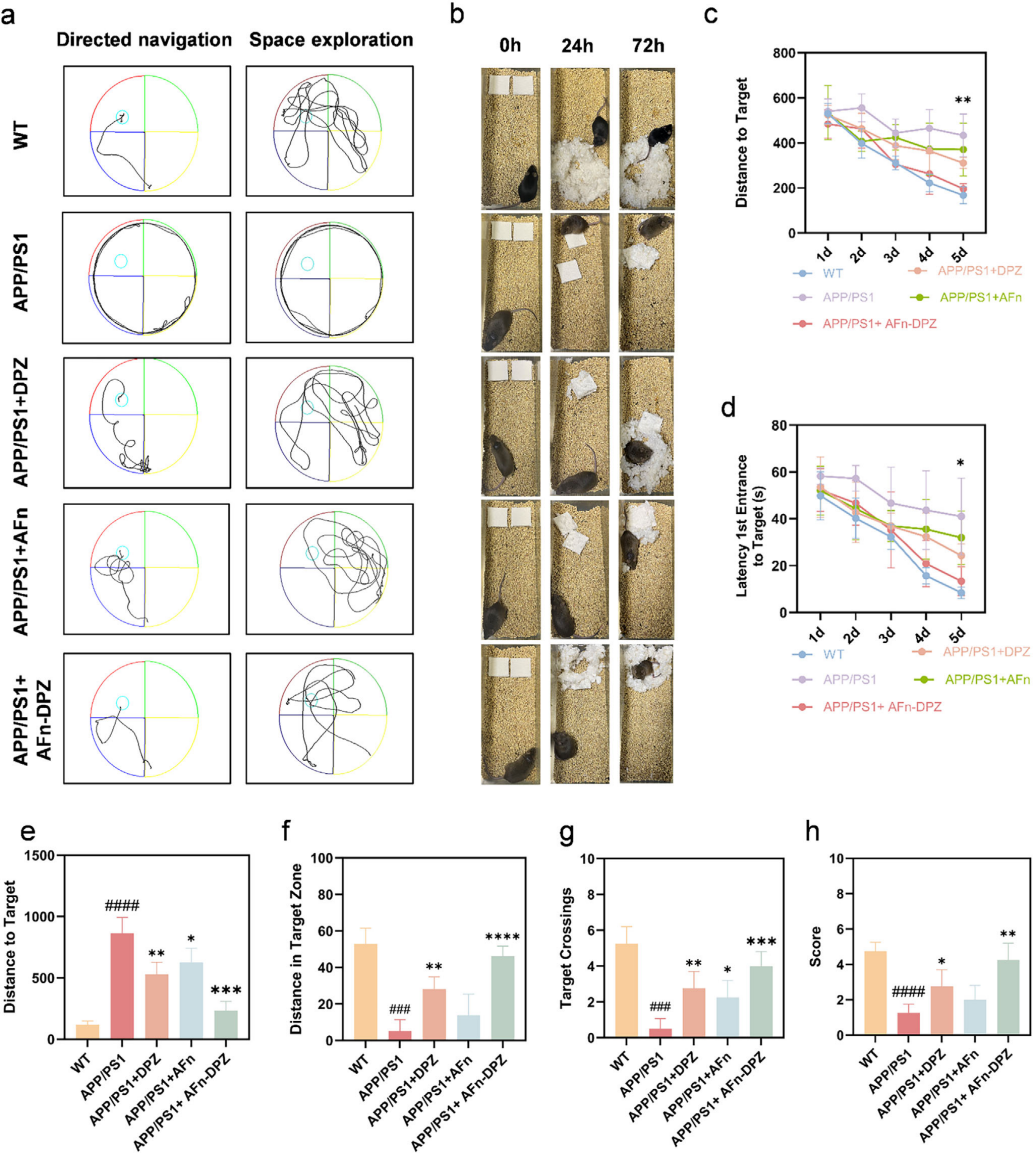
Behavioral evaluation of AD model mice following AFn‐DPZ therapy. (a) Representative swimming trajectories of each group during the orientation navigation and spatial probe trials in the Morris water maze test. (b) Representative images of nest‐building behavior on day 1 and day 3. (c) Path length to the target platform during the first trial across days 1–5 of the orientation navigation phase (n = 4). **p < 0.01 for the APP/PS1‐AFn‐DPZ group compared with the APP/PS1 group. (d) Latency to first reaching the target platform during the first trial across days 1–5 of the orientation navigation phase (n = 4). *p < 0.05 for the APP/PS1‐AFn‐DPZ group compared with the APP/PS1 group. (e) Path length to the target platform during the first trial of the spatial probe phase (n = 4). (f) Time spent in the target quadrant during the spatial probe phase (n = 4). (g) Number of platform crossings during the spatial probe phase (n = 4). (h) Nest‐building scores on day 3 (n = 4). ##p < 0.01, ###p < 0.001, ####p < 0.0001 compared with the WT group; *p < 0.05, **p < 0.01, ***p < 0.001 compared with the APP/PS1 group.

Nesting behavior can be impaired by hippocampal damage and neurodegenerative conditions. Therefore, nesting performance was also evaluated in this study (Figure 6b). In APP/PS1 mice, the Nestlet material showed little change after one day, and while partially shredded after three days, it was not gathered into a nest—indicating disrupted nesting behavior. In contrast, drug‐treated mice, especially those receiving AFn‐DPZ, exhibited extensive shredding of the Nestlet and constructed well‐defined, recognizable nests after three days, leading to significantly improved nest scores (Figure 6h). Consistent with the results from the Morris water maze test, AFn‐DPZ treatment also mitigated cognitive decline.

### 3.5 AFn‐DPZ Restores Brain Function

A hallmark of AD is the presence of abnormal Aβ plaques in the brain [41]. To investigate whether AFn‐DPZ treatment could reduce Aβ plaque deposition, we performed Congo Red staining and immunohistochemistry on brain tissue sections. Numerous Aβ plaques were observed in the hippocampus and cortex of APP/PS1 mice. Treatment with DPZ, AFn, and AFn‐DPZ resulted in varying degrees of reduction in amyloid deposition in these regions (Figure 7a–d, g–j). Notably, mice treated with AFn‐DPZ exhibited a substantial decrease in amyloid plaques in both the hippocampus and cortex, indicating that AFn‐DPZ administration exerts superior efficacy in reducing Aβ plaque burden and ameliorating cognitive and memory impairments in AD compared to other treatments.Furthermore, Nissl staining was conducted to assess the recovery of neuronal damage. Distinct neuronal injury, characterized by cell deformation, pyknosis, and nuclear disappearance, was observed in the hippocampus and cortex of APP/PS1 mice. However, this damage was significantly alleviated following AFn‐DPZ treatment (Figure 7e, f, k, l), suggesting that AFn‐DPZ mitigates neuronal loss.

**FIGURE 7:**
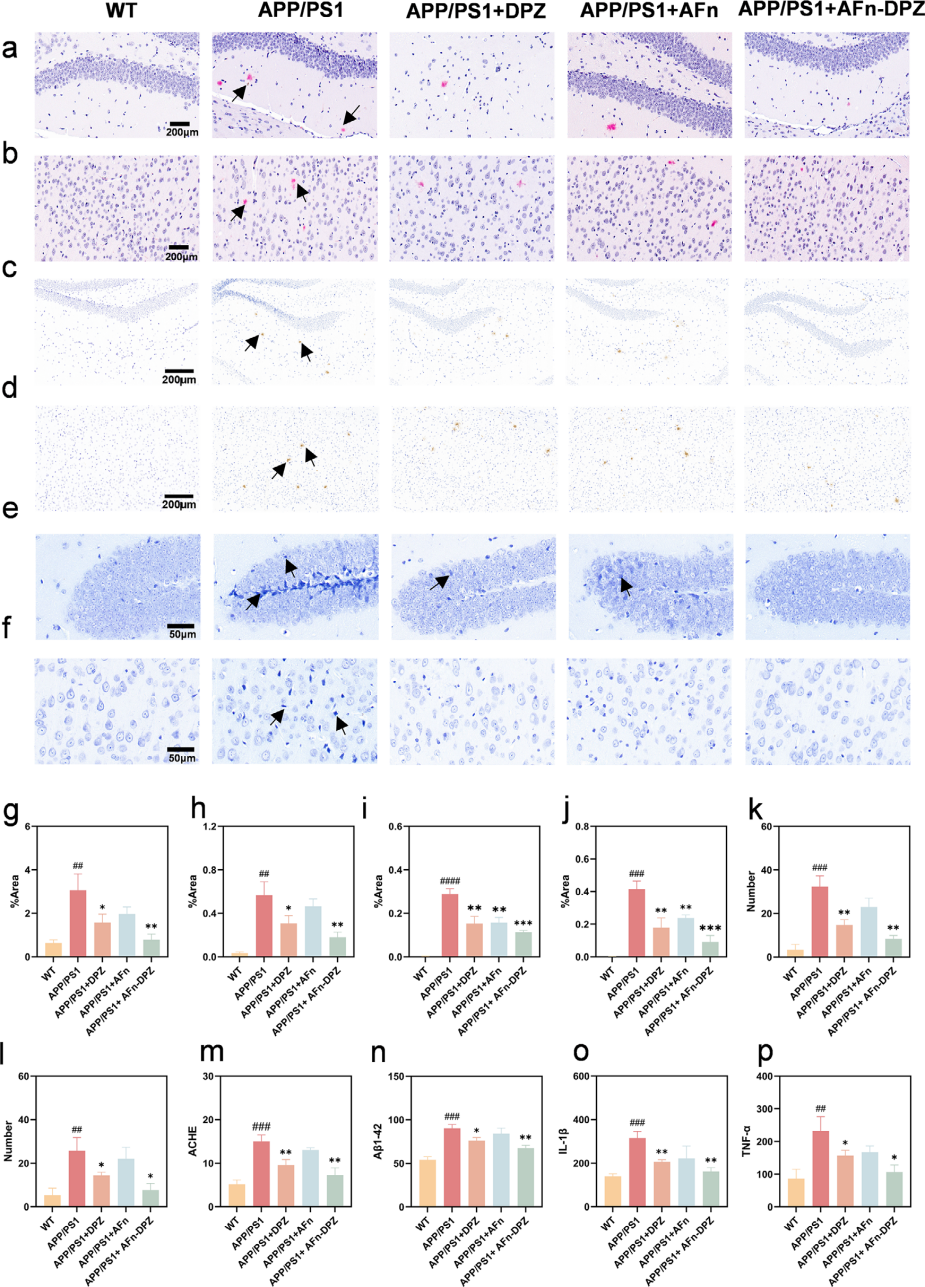
AFn‐DPZ mitigates neuronal injury and restores brain function in vivo.Representative images of amyloid plaques (indicated by black arrows) detected by Congo Red staining in the hippocampus (a) and cortex (b). Immunohistochemical detection of amyloid‐β (indicated by black arrows) in the hippocampus (c) and cortex (d). Nissl staining in the hippocampus (e) and cortex (f) assessing neuronal health via Nissl body integrity. Black arrows indicate pyknotic, shrunken, and necrotic neurons. Quantitative analysis of Congo Red staining area in the hippocampus (g) and cortex (h) (n = 3). Quantitative analysis of amyloid‐β immunohistochemical staining in the hippocampus (i) and cortex (j) (n = 3). Quantitative analysis of pyknotic, shrunken, and necrotic neurons from Nissl staining in the hippocampus (k) and cortex (l) (n = 3). (m) Quantification of AChE levels in hippocampal tissues by ELISA (n = 3). (n) Quantification of Aβ1‐42 levels in hippocampal tissues by ELISA (n = 3). (o) Quantification of IL‐1β levels in hippocampal tissues by ELISA (n = 3). (p) Quantification of TNF‐α levels in hippocampal tissues by ELISA (n = 3). ##p < 0.01, ###p < 0.001 compared with the WT group. *p < 0.05, **p < 0.01, ***p < 0.001 compared with the APP/PS1 group.

DPZ, an AChE inhibitor, is known to improve cognition in patients with mild‐to‐moderate dementia by inhibiting postsynaptic degradation of hippocampal ACh [42]. We evaluated the effect of AFn‐DPZ on AChE reduction using an ELISA kit. Compared with the WT group, AChE levels were significantly elevated in the hippocampus of APP/PS1 mice. In contrast, all treatment groups showed significant downregulation of AChE expression, with AFn‐DPZ demonstrating the most potent effect (Figure 7m). Previous studies indicate that DPZ reduces cerebral Aβ deposition and levels of pro‐inflammatory mediators, thereby ameliorating neuronal death [43]. We assessed representative inflammatory mediators in the hippocampus, including TNF‐α and IL‐1β, and found that their levels were higher in APP/PS1 mice than in WT controls. AFn‐DPZ treatment reduced these pro‐inflammatory cytokines to near‐normal levels (Figure 7n, o). The ELISA results of BDNF protein levels showed that BDNF expression in the APP/PS1 model group was significantly lower than that in the WT control group, indicating reduced neurotrophic support in AD mice. Notably, AFn‐DPZ treatment significantly upregulated BDNF levels in the hippocampus, which were markedly higher than those in the model group, the free DPZ group, and the AFn group (Figure S4a). Finally, we evaluated the effect of AFn‐DPZ on Aβ1‐42 levels using ELISA. Hippocampal Aβ1‐42 was significantly increased in the APP/PS1 group compared to WT controls, whereas all treatment groups exhibited marked downregulation of Aβ1‐42, with AFn‐DPZ showing the strongest reduction (Figure 7p).

Collectively, these findings indicate that AFn‐DPZ exerts neuroprotective effects by inhibiting AChE, alleviating neuroinflammation, and reducing Aβ1‐42 levels. In summary, the results demonstrate that AFn‐DPZ provides superior neuroprotection in vivo compared to other formulations, improving memory deficits and cognitive impairment, and restoring brain function in AD mice.

### 3.6 AFn‐DPZ Ameliorates AD‐Associated Proteomic Network Signatures

Proteomic analysis of the mouse hippocampus revealed that, compared to the APP/PS1 group, the APP/PS1+AFn‐DPZ group exhibited 288 differentially expressed proteins (DEPs), including 216 up‐regulated and 72 down‐regulated proteins (Figure 8a, b). Gene Ontology (GO) enrichment analysis indicated that within biological processes, DEPs were primarily enriched in “negative regulation of endopeptidase activity.” Regarding molecular functions, the DEPs were mainly associated with “serine‐type endopeptidase inhibitor activity” (Figure 8c). This finding is consistent with previous research reporting that glycoproteins captured from the serum of Alzheimer's disease patients are closely linked to serine‐type endopeptidase inhibitor activity and receptor binding [44]. Prolyl oligopeptidase, a large cytosolic serine peptidase widely distributed in the central nervous system—including the hypothalamus, striatum, hippocampus, cortex, and amygdala—has been implicated in cognitive function. Inhibitors of prolyl oligopeptidase have been shown to significantly ameliorate memory impairment and play an important role in regulating learning and memory processes, highlighting their potential as therapeutic agents for Alzheimer's disease [45]. KEGG pathway enrichment analysis further demonstrated that the DEPs were predominantly enriched in the “complement and coagulation cascades” (Figure 8d). Previous studies have reported that chronic cerebral hypoperfusion (CCH), often associated with Alzheimer's disease in the elderly, can impair the clearance of Aβ due to blood‐brain barrier dysfunction, thereby accelerating AD pathology. CCH has been shown to activate the complement and coagulation cascades in the brains of novel AD model mice, accompanied by accelerated AD pathogenesis [46].

**FIGURE 8:**
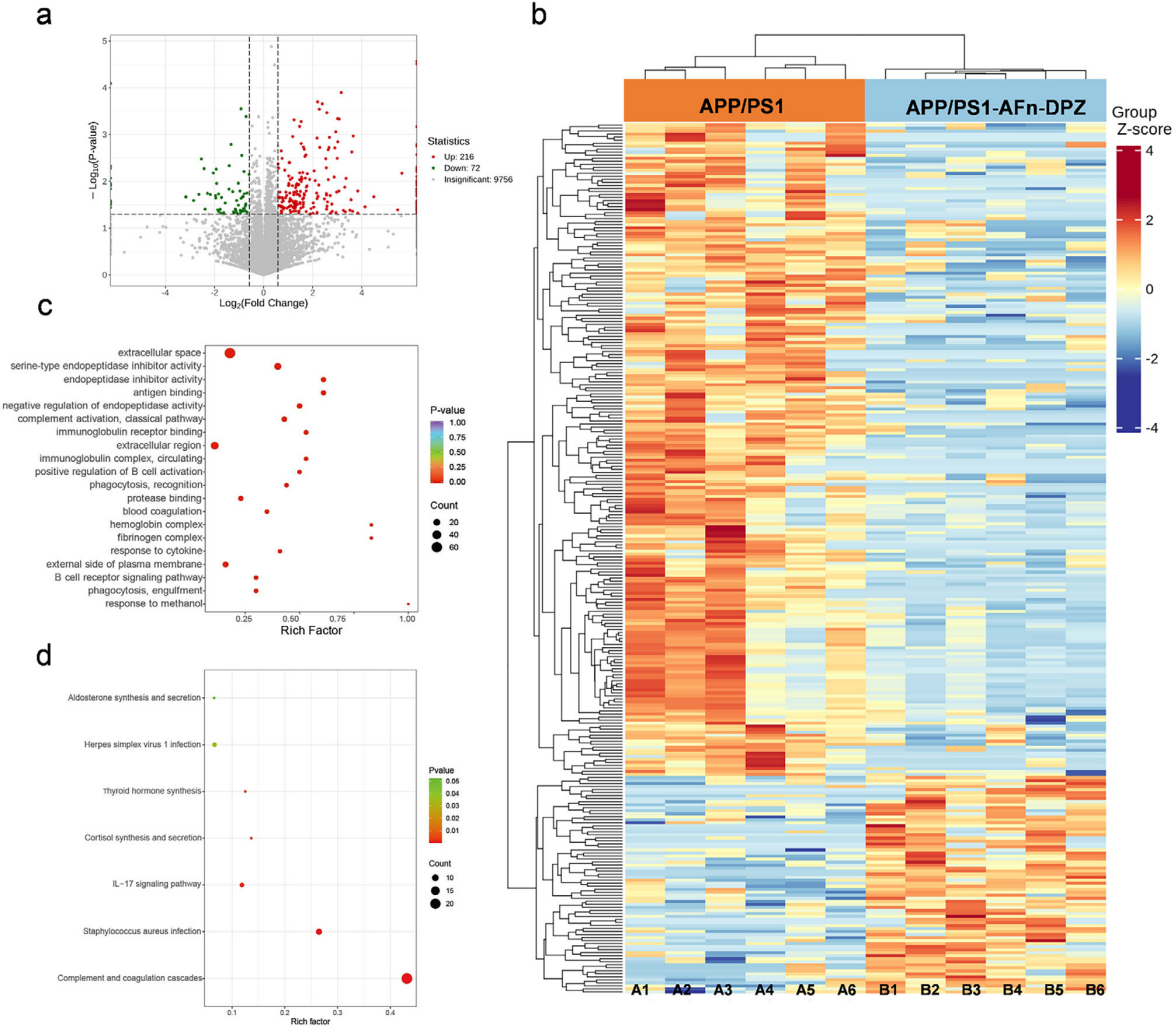
Proteomic changes after the treatment of AFn‐DPZ in the hippocampus of APP/PS1 mice. (a) Volcano plot illustrating DEPs in AFn‐DPZ‐treated APP/PS1 mice compared to untreated APP/PS1 controls. (b) Heatmap displaying the expression profiles of DEPs in APP/PS1 mice before and after AFn‐DPZ treatment. (c) Bubble plot of significantly enriched Gene Ontology (GO) terms for the identified DEPs. (d) KEGG pathway bubble plot showing significant enrichment of DEPs associated with Alzheimer's disease.

### 3.7 In Vivo Biocompatibility

Therapeutic agents for AD must exhibit favorable biocompatibility. We therefore evaluated the biocompatibility profile of AFn‐DPZ in a mouse model. Histopathological examination via H&E staining revealed that tissue sections of the heart, liver, spleen, lung, and kidneys from the AFn‐DPZ‐treated group remained within normal limits compared to the WT group, with no observable evidence of tissue inflammation or injury (Figure 9a). Furthermore, no significant alterations were detected in routine hematological biomarkers or biochemical parameters between the WT and AFn‐DPZ groups (Figure 9b–k), indicating that the AFn‐DPZ nanoformulation did not elicit systemic inflammatory responses following intravenous administration via the tail vein. Collectively, these results confirm that intravenously administered AFn‐DPZ possesses desirable biocompatibility, supporting its safety profile for AD treatment.

**FIGURE 9:**
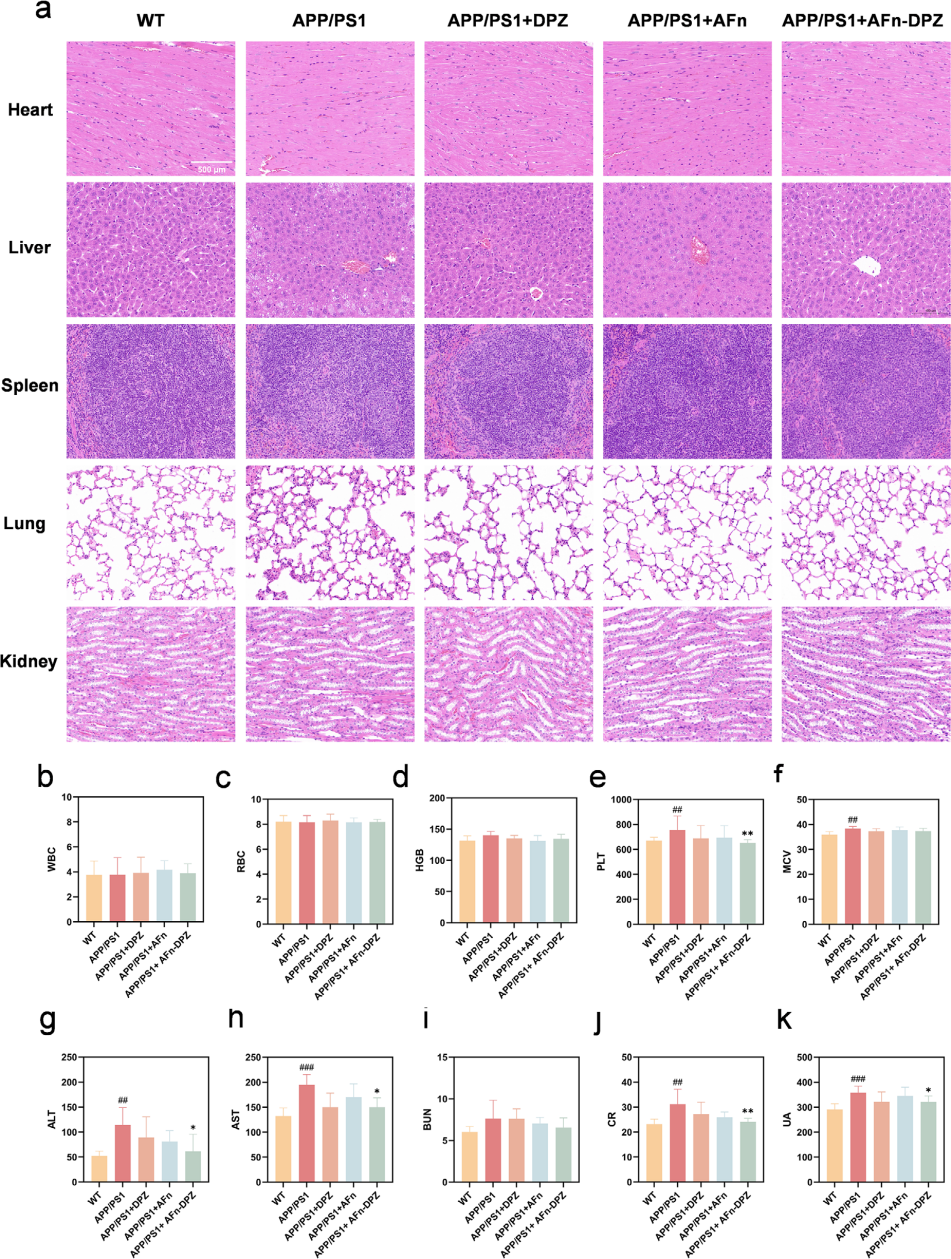
In vivo biocompatibility of AFn‐DPZ. (a) Representative images of hematoxylin and eosin (H&E)‐stained sections of the heart, liver, spleen, lungs, and kidneys from each treatment group. (b‐k) Results of complete blood panel and hepatic/kidney function tests for all experimental groups (n = 6).

## 4 Discussion

In this study, we successfully constructed an AFn‐based brain‐targeted nanodelivery system, designated AFn‐DPZ. Compared with a simple physical mixture of AFn and DPZ, the AFn‐DPZ nanodelivery system constructed in this study offers irreplaceable and unique advantages. First, at the structural level, simple physical mixing cannot effectively encapsulate DPZ within the cage‐like cavity of AFn, and the drug lacks stable association with the carrier, leading to rapid metabolic clearance of DPZ upon in vivo exposure and difficulty in maintaining effective drug concentrations in the brain. In contrast, AFn‐DPZ achieves efficient DPZ encapsulation through pH‐mediated reassembly, remains stable during blood circulation, and crosses the BBB by leveraging the inherent brain‐targeting property of AFn. Second, at the functional level, the physical mixture cannot reproduce the receptor‐mediated transcytosis process of AFn‐DPZ, and therefore cannot induce EphB1‐related cytoskeletal rearrangement or transient BBB opening, nor can it achieve the brain‐targeting efficiency and sustained‐release behavior unique to the nanoformulation. The in vivo distribution, pharmacodynamic, and proteomic data from this study collectively demonstrate that only when DPZ is encapsulated within the AFn cavity can its brain accumulation be significantly increased, its duration of action prolonged, and multiple therapeutic effects such as anti‐Aβ deposition, neuroinflammation reduction, and cognitive improvement be synergistically achieved. Therefore, the AFn‐DPZ nanodelivery system not only overcomes the inherent limitations of physical mixtures in terms of stability and targeting but also provides a platform with greater clinical translation potential for the precision treatment of central nervous system disorders.

This study validated its transport mechanism across the BBB mediated by the EphB1 receptor, as well as its efficacy in alleviating Alzheimer's disease pathology and cognitive impairment. In contrast to the commonly studied TfR pathway, we identified and confirmed for the first time that EphB1 is a specific binding target of AFn on brain microvascular endothelial cells. This finding not only provides a new perspective for understanding the BBB transmembrane transport mechanism of ferritin family proteins but also establishes a molecular foundation for developing EphB1‐based brain‐targeted delivery strategies.

First, regarding the mechanism of BBB transport, previous studies have generally suggested that ferritin enters the brain primarily through TfR1‐mediated transcytosis. However, Yan et al. [30]. demonstrated that AFn lacks binding affinity for murine TfR1, suggesting the existence of an alternative receptor. Our molecular fishing, mass spectrometry, and molecular docking results unequivocally identified EphB1 as a specific binding partner of AFn. Furthermore, using EphB1 competitive inhibition experiments, we confirmed that blocking EphB1 significantly reduces AFn transport across the BBB, providing causal evidence for the necessity of the AFn–EphB1 interaction. Notably, binding of AFn to EphB1 induced transient and reversible cytoskeletal rearrangement and decreased tight junction integrity.

Among the differential proteins identified by proteomics, keratin type I cytoskeletal 19 (K19), immunoglobulin heavy constant mu (IgM), and alpha‐2‐macroglobulin‐like protein 1 (A2M‐like 1) may be potentially associated with EphB1‐mediated BBB regulation. As a type I keratin intermediate filament, K19 maintains the spread morphology of cells by stabilizing the actin network [47]. Activation of the RhoA/ROCK pathway can downregulate K19 expression, reduce cytoskeletal rigidity, and synergistically promote intercellular gap formation [48]. Our proteomic analysis revealed downregulation of K19 following AFn‐DPZ treatment, which may represent a consequence of RhoA/ROCK activation. In addition, EphB receptors can indirectly regulate RhoA activity by degrading the RhoA GEF Ephexin5 [34]. Therefore, K19 may serve as a downstream effector molecule of the EphB1–RhoA signaling axis, providing a structural basis for facilitating BBB drug delivery. The detection of the IgM constant chain suggests enhanced trans‐barrier transport of macromolecular proteins following BBB permeability changes [49], which may be favorable for the brain delivery of therapeutic agents such as donepezil. A2M‐like 1, a broad‐spectrum protease inhibitor, may play a neuroprotective role during drug transport across the BBB by mitigating potential damage resulting from protease cascade reactions [50]. The coordinated changes of these proteins preliminarily indicate that an EphB1‐targeting strategy may promote the brain delivery of donepezil while maintaining a relatively controlled trans‐barrier window, offering proteomic insights for targeted therapy of AD.

Second, regarding the multi‐target synergy of therapeutic efficacy, AFn‐DPZ exhibited significantly better therapeutic effects than free DPZ in an AD mouse model, which can be attributed to its efficient brain‐targeted delivery and sustained‐release properties. LC‐MS/MS results showed that, at the brain tissue level, the AFn‐DPZ group exhibited higher drug concentrations than the free DPZ group, indicating the superior brain delivery efficiency of AFn. At the plasma level, no significant difference in drug concentration was observed between the free DPZ group and the AFn‐DPZ group, suggesting that free DPZ, failing to enter the brain, remains in the peripheral circulation, where it is delivered to metabolic and excretory organs and rapidly cleared. Collectively, these findings demonstrate that AFn, as a delivery carrier, can optimize the in vivo pharmacokinetic behavior of DPZ, enhance its brain‐targeting capability, improve its therapeutic efficacy in the brain, and potentially reduce the adverse effects associated with peripheral drug exposure. This directly translated into enhanced AChE inhibition in the brain tissue and strengthened cholinergic neurotransmission. Notably, AFn‐DPZ also exhibited neuroprotective effects beyond the single pharmacological action of DPZ: it reduced Aβ deposition, alleviated neuroinflammation (IL‐1β, TNF‐α), decreased oxidative stress (ROS), restored mitochondrial function (MMP recovery), and upregulated BDNF expression. This “delivery + therapy” dual functionality distinguishes AFn‐DPZ from conventional nanocarriers and highlights its unique advantages in the treatment of complex neurodegenerative diseases.

Third, with respect to clinical translation potential and advantages, current mainstream brain‐targeted nanodelivery systems primarily include liposomes, polymer nanoparticles (e.g., PLGA), solid lipid nanoparticles, inorganic nanoparticles (e.g., gold nanoparticles, magnetic nanoparticles), and natural protein nanocages (e.g., ferritin) [51, 52]. The preparation of polymer nanoparticles or liposomes often involves organic solvents, emulsifiers, or high‐pressure homogenization, carrying risks of solvent residue and yielding relatively large particle sizes [53]. Degradation products of polymer nanoparticles such as PLGA (lactic acid and glycolic acid) can cause local sterile inflammation at high concentrations [54]. In contrast, inorganic nanoparticles (e.g., gold and magnetic particles) are non‐degradable in vivo and rely on slow renal clearance, raising concerns regarding long‐term safety [55]. Moreover, many synthetic nanoparticles (e.g., certain liposomes or unmodified polymer nanoparticles) suffer from drug burst release or short circulation time [53]. AFn‐DPZ possesses the following translational advantages: first, high biocompatibility, as AFn is an endogenous protein that can be metabolized normally by the body with no toxicity from degradation products and no risk of in vivo accumulation; second, it circumvents the safety hazards associated with organic solvents and complex manufacturing processes; third, it achieves brain targeting without the need for additional chemical modification; and fourth, it has broad applicability, as this strategy is not limited to AD but may also hold potential for other CNS disorders involving BBB dysfunction or upregulated EphB1 expression, such as glioma, Parkinson's disease, and traumatic brain injury.

Finally, regarding limitations. 1) To demonstrate the advantage of the nanoparticle formulation, a physical mixture of AFn and DPZ should ideally be included as a control. However, when DPZ is physically mixed with AFn, the resulting formulation has a pH of approximately 6.0. This acidic environment could potentially affect systemic acid–base balance in vivo. More importantly, adjusting the pH to 7.4 to mimic physiological conditions leads to precipitation of donepezil hydrochloride due to its poor solubility at neutral pH, rendering the physical mixture unstable and unsuitable for administration. Therefore, referring to previous study designs [56, 57, 58], we adopted an alternative experimental framework using separate administration groups instead of a physical mixture.

2) The sample size (n = 4 per group) is relatively small. This decision was guided by the ethical principle of Reduction (one of the 3Rs—Replacement, Reduction, Refinement), which seeks to minimize animal use while still obtaining scientifically meaningful data. To ensure the reasonableness of this sample size, we reviewed the literature prior to conducting the experiments. Several studies published in reputable journals have employed a similar or even identical sample size. For instance, Kedia et al. [59]. used n = 4 per group for behavioral assessments, and Liu et al. [60]. reported n = 4–6 per group in comparable experimental settings. Collectively, these examples demonstrate that a sample size of 4–6 per group is reasonable and widely accepted in exploratory studies where effect sizes are substantial and the 3R principle is prioritized. Nevertheless, further validation with a larger sample size should be conducted to strengthen the statistical power and generalizability of our conclusions.

3) Although we have demonstrated the importance of the AFn–EphB1 interaction through in vitro and in vivo experiments, the essential requirement of this pathway has not yet been validated at the genetic level (e.g., using EphB1 conditional knockout mice). Furthermore, the downstream signaling pathways following EphB1 activation, such as RhoA/ROCK‐mediated cytoskeletal rearrangement, remain to be fully elucidated. The sample size in this study is relatively limited, and larger‐scale, multi‐center preclinical studies are needed in the future, along with further evaluation of the safety of long‐term, repeated dosing.

4) Additionally, a certain reduction in amyloid plaque burden in the hippocampus and cortex of APP/PS1 mice was also observed in the empty AFn carrier control group (without DPZ encapsulation). This phenomenon may be related to the intrinsic bioactivity of ferritin itself. As a natural iron‐chelating protein, ferritin possesses catalase‐like activity, potentially acting by scavenging intracellular reactive oxygen species, regulating iron homeostasis, and attenuating lipid peroxidation [61], which may indirectly affect amyloid precursor protein metabolism and the oxidative stress microenvironment promoting Aβ aggregation. However, the primary objective of this study was to validate the brain‐targeted delivery and synergistic therapeutic effect of AFn‐DPZ, and the current experimental design does not fully dissociate the physical delivery function of the AFn carrier from its inherent antioxidant effects. Whether the inhibitory effect of AFn alone on Aβ deposition persists under longer treatment regimens and whether it indirectly affects Aβ metabolism through the EphB1 signaling pathway require further investigation. Therefore, when interpreting the therapeutic effects of AFn‐DPZ, a modest direct contribution of the AFn carrier itself to AD pathology cannot be completely excluded. Follow‑up studies should include an AFn‐only group and extend the observation period to more clearly delineate the respective roles of the carrier and the drug.

5) Although AFn‐DPZ has shown encouraging brain‐targeted delivery efficiency and therapeutic effects in animal models, its large‐scale production process has not yet been established and remains at the laboratory research stage. Future efforts should focus on developing a Good Manufacturing Practice (GMP)‐compliant large‐scale production process to facilitate the clinical translation of this nanoformulation.

## 5 Conclusion

After iron removal, ferritin formed a hollow cage‐like structure capable of encapsulating DPZ, leading to the successful construction of the AFn‐DPZ nanodrug delivery system. This formulation is characterized by small particle size, high stability, excellent sustained‐release properties, and good biocompatibility.

AFn‐DPZ specifically binds to the EphB1 receptor on the surface of brain microvascular endothelial cells, inducing cytoskeletal rearrangement and reducing tight junctions, thereby facilitating efficient brain entry and subsequent neuronal uptake. This process enhances the brain‐targeted delivery efficiency of DPZ and its bioavailability in the brain.

AFn‐DPZ alleviates Aβ_25_‑_35_‐induced neurotoxicity and improves mitochondrial function in Aβ_25_‑_35_‐damaged neurons, thereby reducing neuronal apoptosis, decreasing ROS production, and mitigating oxidative stress.

AFn‐DPZ accumulates in the brains of APP/PS1 mice and improves their cognitive performance at the behavioral level. It effectively reduces Aβ expression and amyloid plaque burden in the hippocampus and cortex, decreases neuronal loss, increases the number of Nissl bodies, maintains normal neuronal function, lowers hippocampal AChE levels, reduces hippocampal inflammatory cytokine levels, alleviates neuroinflammation, and upregulates BDNF expression. Its proteomic profile is highly correlated with key pathological pathways in Alzheimer's disease, including the regulation of endopeptidase activity and the complement/coagulation cascade.

Moreover, administration of AFn‐DPZ does not induce obvious hepatorenal toxicity or immunosuppressive responses in normal mice.

In summary, the AFn‐DPZ nanodrug delivery system enables efficient brain targeting and multi‐target intervention of AD pathological processes, demonstrating potential clinical application value in improving AD‐related cognitive impairment.

## Author Contributions

**Shi‐Lin Wen**: Writing – original draft, Data curation. **Jing‐Jing Gao**: Writing – original draft, Validation. **Zhi‐Xian Wang**: Writing – original draft. **Qiu‐min Ma**: Visualization. **Xiao‐Ling Xu**: Writing – review & editing, Conceptualization. **Jianer Chen**: Writing – review & editing,Supervision,Resources.

## Funding

This study was supported by the Scientific research project of Zhejiang Rehabilitation Medical Center (ZKJC2203), Construct Program of the Key Discipline in Zhejiang Rehabilitation Medical Center (ZKXK02), the Key Scientific and Technological Research and Development Program of Zhejiang Province (2021C03050), the National Natural Science Foundation of China (82372109).

## Declaration of AI use

During the preparation of this manuscript, no machine‐generated or AI‐assisted content was used. All writing, data analysis, and figure preparation were completed solely by the authors and their research team.

## Conflicts of Interest

The authors declare no conflicts of interest.

## Supporting information




**Supporting File**: advs76480‐sup‐0001‐SuppMat.docx.

## Data Availability

The data that support the findings of this study are available from the corresponding author upon reasonable request.
