# Supplementary material for: EphB1‐Mediated Transient Blood‐Brain Barrier Opening Facilitates a Ferritin‐Based Nanotherapeutic for Alzheimer's Disease
Source: Adv Sci (Weinh). 2026 Jul 6;13(55):e76480. doi: 10.1002/advs.76480 (PMC13335922; doi:10.1002/advs.76480)
Supplement: Supplementary file 1 [file ADVS-13-e76480-s001.docx]

**EphB1-Mediated Transient Blood-Brain Barrier Opening Facilitates a Ferritin-Based Nanotherapeutic for Alzheimer's Disease**

Shilin Wen^a, b, c, d, #^, Jingjing Gao^a, b, c, #^, Zhixian Wang ^e, #^, Qiumin Ma ^a, b, c^, Xiao-Ling Xu^b,^ *, Jianer Chen^a, c, d,^ *

^a^ Department of Rehabilitation, The Third Affiliated Hospital of Zhejiang Chinese Medical University, Hangzhou, Zhejiang, China;

^b^ Shulan International Medical College, Zhejiang Shuren University, Hangzhou, China;

^c^ The Third Clinical Medical College, Zhejiang Chinese Medical University, Hangzhou, China;

^d^ Department of Neurorehabilitation, Zhejiang Rehabilitation Medical Center, Hangzhou, China;

^e^ Department of Rehabilitation, Affiliated Nanhua Hospital, University of South China, Hunan Province, Hengyang, China

^#^ These authors contributed equally to this work.

*Corresponding Author

Correspondence:

Jianer Chen, Email chenje@zcmu.edu.cn;

Xiao-Ling Xu, Email ziyao1988@zju.edu.cn

**Supplementary experimental section**

**In vitro BBB drug permeability assay**

An in vitro BBB model was established using bEnd.3 cells cultured on Transwell inserts. The TEER value was monitored daily using a Millicell ERS meter. After the TEER value stabilized, equal amounts of free DPZ and AFn-DPZ formulation were added to the upper chamber, and the cells were further incubated at 37°C in a 5% CO₂ incubator for 3 h. The culture medium in the lower chamber was then collected. A 10 µL aliquot of the medium sample was mixed with 1 µL of 0.1 M NaOH for alkalization, followed by extraction with 100 µL of ethyl acetate. After vortexing and centrifugation at 10,000 rpm for 5 min, the supernatant was collected, concentrated to dryness by centrifugation, and reconstituted in 100 µL of 50% methanol in water. The concentration of DPZ in the lower chamber medium was then determined by LC-MS/MS to evaluate the permeability coefficients of different formulations across the in vitro BBB.

**Measurement of BDNF levels by ELISA**

The protein expression level of brain-derived neurotrophic factor (BDNF) in brain tissue was measured using an ELISA. Mouse hippocampal tissues were harvested and homogenized in RIPA lysis buffer containing protease inhibitors. After centrifugation at 4°C, the supernatant was collected. The assay was performed according to the manufacturer‘s instructions of the BDNF ELISA kit. Briefly, standards and samples were added to a microplate pre-coated with capture antibody, incubated, and washed. A detection antibody and horseradish peroxidase (HRP)-conjugated secondary antibody were then added, followed by substrate solution for color development. The absorbance was measured at 450 nm using a microplate reader. The BDNF concentration was calculated from a standard curve and normalized to total protein content.

**Time-dependent changes in TEER values after AFn addition in an in vitro BBB model**

An in vitro BBB model was established using bEnd.3 cells cultured on Transwell inserts. After TEER values stabilized, AFn was added to the upper chamber. TEER values were measured using a Millicell ERS meter at 0, 1, and 3 h after AFn addition. Before each measurement, the insert was equilibrated at room temperature for 15 min. Three replicate measurements were taken per well and averaged. Calculate the TEER value to evaluate the transient and time-dependent effects of AFn on BBB integrity.

**EphB1 competitive inhibition assay**

To verify that AFn crosses the BBB via EphB1 receptor-mediated transport, an in vitro BBB model was established using bEnd.3 cells cultured on Transwell inserts. After TEER values stabilized, the cells in the upper chamber were pre-incubated with 220 nM EphB1-specific inhibitor (EphB1-IN-1) at 37°C for 30 min, followed by the addition of FITC-labeled AFn (AFn-FITC) for further incubation for 3 h. The fluorescence intensity in the medium from the lower chamber was measured using a fluorescence microplate reader.

**Preparation of plasma samples and determination by LC-MS/MS**

A 10 µL aliquot of the plasma sample was mixed with 1 µL of 0.1 M NaOH for alkalization, followed by extraction with 100 µL of ethyl acetate. After vortexing and centrifugation at 10,000 rpm for 5 min, the supernatant was collected, concentrated to dryness by centrifugation, and reconstituted in 100 µL of 50% methanol in water prior to LC-MS/MS analysis.

a

**Figure S1.** (a) Encapsulation efficiency and drug loading capacity of AFn-DPZ.

**Figure S2.** (a) Fluorescence quantitative analysis of the tight junction protein occludin in bEnd.3 cells after incubation with AFn for 1h and 3h (n=3), *p < 0.05, ***p < 0.001, ****p < 0.0001. (b) TEER values measured at 1 h and 3 h after AFn addition in the in vitro BBB model (n=3), **p < 0.01***p < 0.001. (c) The fluorescence intensity in the lower chamber after AFn-FITC addition to the upper chamber of the in vitro BBB model with EphB1 inhibitor intervention (n=3), **p < 0.01.

a

**Figure S3.** (a) Permeability coefficient of DPZ and AFn-DPZ across the in vitro BBB (n=3), ****p < 0.0001.

a

**Figure S4.** (a) Quantification of BDNF levels in hippocampal tissues by ELISA (n=3), ##p < 0.01 compared with the WT group. *p < 0.05, **p < 0.01, compared with the APP/PS1 group.

**Table 1** DPZ concentrations in the brains of APP/PS1 mice at different time points.

| Group  (n=3) |  | Concentration(ng/g) | | | |
| --- | --- | --- | --- | --- | --- |
|  | 0.25h | 2h | 3h |  | 24h |
| DPZ | 945.36±55.04 | 80.22±1.61 | 33.45±3.16 |  | 1.29±0.23 |
| AFn-DPZ | 2870.03±38.77^****^ | 178.25±11.31^***^ | 51.05±3.40^**^ |  | 1.79±0.44 |

**p < 0.01, ***p < 0.001, ****p < 0.0001 compared with the DPZ group.

**Table 2** DPZ concentrations in the plasma of APP/PS1 mice at different time points.

| Group  (n=3) |  | Concentration(ng/mL) | | | | |
| --- | --- | --- | --- | --- | --- | --- |
|  | 0.25h | | 0.5h | 2h | 12h | 24h |
| DPZ | 45.10±13.09 | | 28.07±6.03 | 3.10±1.56 | 2.63±2.52 | 0.77±0.88 |
| AFn-DPZ | 29.47±23.09 | | 16.50±1.96 | 2.93±1.89 | 1.17±0.33 | 0.80±0.37 |
